# GLUT1-mediated microglial proinflammatory activation contributes to the development of stress-induced spatial learning and memory dysfunction in mice

**DOI:** 10.1186/s13578-024-01229-1

**Published:** 2024-04-16

**Authors:** Xue Wang, Yuhan Wu, Yingrui Tian, Hui Hu, Yun Zhao, Binghua Xue, Zhaowei Sun, Aijun Wei, Fang Xie, Ling-Jia Qian

**Affiliations:** 1 Beijing Institute of Basic Medical Sciences, Academy of Military Medical Sciences, #27 Taiping Road, Haidian, Beijing, 100850 China; 2 Centers for Disease Control and Prevention, Jiulongpo District, Chongqing, 400050 China

**Keywords:** Chronic stress, Microglia, Proinflammatory activation, GLUT1, Spatial memory dysfunction

## Abstract

**Background:**

Stress is a recognized risk factor for cognitive decline, which triggers neuroinflammation involving microglial activation. However, the specific mechanism for microglial activation under stress and affects learning and memory remains unclear.

**Methods:**

The chronic stress mouse model was utilized to explore the relationship between microglial activation and spatial memory impairment. The effect of hippocampal hyperglycemia on microglial activation was evaluated through hippocampal glucose-infusion and the incubation of BV2 cells with high glucose. The gain-and loss-of-function experiments were conducted to investigate the role of GLUT1 in microglial proinflammatory activation. An adeno-associated virus (AAV) was employed to specifically knockdown of GLUT1 in hippocampal microglia to assess its impact on stressed-mice.

**Results:**

Herein, we found that chronic stress induced remarkable hippocampal microglial proinflammatory activation and neuroinflammation, which were involved in the development of stress-related spatial learning and memory impairment. Mechanistically, elevated hippocampal glucose level post-stress was revealed to be a key regulator of proinflammatory microglial activation via specifically increasing the expression of microglial GLUT1. GLUT1 overexpression promoted microglial proinflammatory phenotype while inhibiting GLUT1 function mitigated this effect under high glucose. Furthermore, specific downregulation of hippocampal microglial GLUT1 in stressed-mice relieved microglial proinflammatory activation, neuroinflammation, and spatial learning and memory injury. Finally, the NF-κB signaling pathway was demonstrated to be involved in the regulatory effect of GLUT1 on microglia.

**Conclusions:**

We demonstrate that elevated glucose and GLUT1 expression induce microglia proinflammatory activation, contributing to stress-associated spatial memory dysfunction. These findings highlight significant interplay between metabolism and inflammation, presenting a possible therapeutic target for stress-related cognitive disorders.

**Supplementary Information:**

The online version contains supplementary material available at 10.1186/s13578-024-01229-1.

## Background

Numerous epidemiological investigations and experimental studies have shown a negative correlation between chronic stress and cognitive function in mammals. Chronic stress has been recognized as an important risk factor leading to cognitive impairment and even dementia [1–3]. The hippocampus, predominantly responsible for spatial learning and memory, is one of the brain’s most vulnerable regions in response to stress [4]. Our previous studies and many others have revealed that chronic stress induces hippocampal structural remodeling and functional impairments, including reduced hippocampal volume, altered synaptic plasticity, decreased neurogenesis, and increased dendritic atrophy, all involved in stress-related learning and memory deficits [5–7]. However, the underlying cellular and molecular mechanisms remain unclear and must be elucidated to prevent and ultimately treat stress-induced cognitive impairment.

Increasing evidence indicates that inflammation is highly associated with the pathogenesis of stress-associated disorders [8]. Immune cell activation and inflammatory factor release in the brain have been observed in chronic stress [9]. Microglia are the most important innate immune cells in the brain and the main source of stress-induced inflammatory molecules that regulate neuroinflammation [10]. Under physiological conditions, microglia are in a “resting” state, and survey the surrounding neurons for cellular debris, performing necessary maintenance [11]. Nevertheless, microglia can be activated and display deleterious or beneficial phenotypes in response to internal or external stresses [12]. The two main microglial phenotypes are the proinflammatory phenotype and the anti-inflammatory phenotype. Studies have shown that proinflammatory activated microglia release proinflammatory cytokines interleukin-6 (IL-6), interleukin-1β (IL-1β), and tumor-necrosis factor-α (TNF-α), contributing to the negative consequences of chronic stress [13]. Considering the important roles of inflammation and microglia in the stress response, targeting microglia may be a novel strategy for addressing chronic stress-induced learning and memory impairment.

The regulation of microglia activation is highly complex, and knowledge of the mechanism underlying chronic stress-induced microglial overactivation is limited. Brain cells use glucose as their primary energetic substrate to support their functions. Glucose supply and utilization in the brain are closely related to many neurobehavioral functions, including learning and memory [14, 15]. However, abnormally elevated glucose levels can have damaging effects on the brain. Recent research has revealed that chronic social defeat stress in adult male mice induces hyperglycemia and disturbs glucose metabolism peripherally and in the brain, directly impairing spatial memory performance [16]. Glucose is taken up by cells via a family of facilitative glucose transporters (GLUTs). Among them, GLUT1 and GLUT3 are the main glucose transporters expressed in the brain and have been found to play critical roles in neural cell function [17]. In addition, GLUT1 is the primary glucose transporter in immune cells, and its role in immunology has recently attracted considerable attention. Studies have shown that the upregulation of GLUT1 expression drives the proinflammatory macrophage phenotype [18]. Nevertheless, whether GLUTs are involved in chronic stress-induced microglial activation and learning and memory impairment remains unclear.

Thus, the present study’s focus was to unravel the effect of chronic stress on microglia activation and the underlying mechanism. We found that chronic unpredictable mild stress (CUMS) induced the microglial proinflammatory phenotype, which participated in the mediation of learning and memory decline. Furthermore, disordered glucose metabolism was demonstrated to play a key role in the above changes by promoting or antagonizing the function of GLUT1 in microglia. Our results provide a potential new target for the treatment of stress-associated cognitive disorders.

## Materials and methods

### Animals

Male C57BL/6J mice (4‑6 weeks old) purchased from SiPeiFu biotechnology company (Beijing, China) were used and randomly assigned to different groups. Mice were exposed to a 12-h/12-h light/dark cycle and had free access to pure water and food except during the stress intervention and behavioral experiments. All of the animal experiments were approved by the Institutional Animal Care and Use Committee of the Academy of Military Medicine Sciences (Permit No: IACUC-DWZX-2021-695) and were in accordance with the National Research Council’s Guide for the Care and Use of Laboratory Animals (8th edition). We made all efforts to minimize animal suffering.

### Chronic unpredictable mild stress procedure

CUMS is a common paradigm used to mimic long-term negative stress both physically and psychologically [19]. In this study, CUMS was used to simulate the effects of long-term negative stimulation in mice according to previous studies [20] with some modifications. The stimulation procedures were as follows: cage tilting at 45° for 12 h, wet bedding for 12 h, continuous lighting for 24 h, food or water deprivation for 12 h, physical restriction for 8 h, forced swimming in 4 °C water for 3 min, shaking at 120 rpm for 30 min. The stimulation methods were randomly scheduled for an 8 weeks period.

### Morris water maze (MWM)

Spatial learning and memory of mice tested by the MWM was performed according to a previous protocol [21]. MWM test was carried out in a circular tank filled with water (diameter 120 cm, height 30 cm, made opaque by adding titanium dioxide, maintained at 25°C) in a room with fixed environment. The maze was artificially divided into four equal quadrants and the platform (10 cm in diameter), which was fixed 1 cm beneath the water surface, was placed in the center of the target quadrant. During the training period, the mice were allowed to swim freely for 60 s to find the platform. Mice failed to find the platform were guided toward it and allowed to stay there for 30 s. Mice were trained twice per day for a 6 days period. At 24 h after the last training trial, the platform was removed, and the mice were tested for memory retention in a probe trial. The swimming path, latency to enter the platform (Latency to platform), swimming distance of first time to enter the platform (Distance to platform), the number of entries to the area of the platform (Platform crossings), and the average swimming speed were recorded and analyzed by Labmaze Animal Behavior Analysis Software V3.2 (Zhongshi Dichuang, Beijing, China).

### Minocycline treatment

Minocycline (MedChemExpress, New Jersey, USA) was used as an inhibitor of microglial activation to investigate the potential involvement of microglial activation in CUMS-induced cognitive decline. Mice were intraperitoneally injected with minocycline (30 mg/kg, diluted in saline) once a week during the last 4 weeks of CUMS procedure (weeks 5–8 of CUMS).

### Stereotaxic surgery for hippocampal cannulation and glucose infusion

The stereotaxic surgery for hippocampal cannulation and glucose infusion were performed as previously described [16]. Mice were anesthetized in an isoflurane-filled box, and secured in a stereotaxic frame (ZhongShi DiChuang, Beijing, China) under gas anesthesia (2% isoflurane in O2 (4 L/min)). The skin on the top of the skull was shaved, disinfected, and then exposed through a longitudinal incision. Four small holes were drilled: two for the implantation of stainless steel cannulas (Plastics One, Wallingford Connecticut, USA) targeted at the hippocampus based on stereotaxic coordinates (-2.46 mm caudal to bregma, ± 1.7 mm from midline, 2.0 mm deep from the skull), and two for the insertion of anchoring screws. The cannulas were fixed using dental acrylic cement, and the wound was closed with stitches. After recovered for 1 w, the mice were inserted with injectors and infused with 0.3 µL per side of either a glucose solution (10 g/L) or saline at the speed of 0.2 µL/min for three consecutive days. The concentration of infused glucose solution was calculated based on a previous study [16], which involved the average hippocampal glucose concentrations observed in CUMS-exposed mice using ELISA, the molecular weight of glucose (180 g/mol), and the expectation of reaching about 10 mg of hippocampal tissue per side with the infusions. Twenty-four hours after the final glucose infusion, the mice were sacrificed.

### Isolation of microglia from mouse hippocampus

Animals were sacrificed by cervical dislocation, and the hippocampi were promptly dissected from the brains of the control mice and CUMS mice, followed by a prechilled PBS wash. Tissue dissociation was performed based on established protocol [22] with minor modifications. In brief, the tissues were digested with Papain (2 mg/mL) (Worthington, Colorado, USA) in RPMI 1640 medium at 37°C for 30 min. The dispersed cells were then passed through a 70 μm nylon mesh and collected through centrifugation at 300 g for 6 min at 4°C. After removing red blood cells using Red Blood Cell Lysis Buffer (Solarbio, Beijing, China) added with 10 µL DNase I (Sigma, Michigan, USA), total cell pellets were resuspended with PBS followed by microglia isolation. Microglia were isolated using anti-CD11b-coated MicroBeads (Miltenyi Biotec, Bergisch Gladbach, Germany) with a MACS multi-stand separator as per the manufacturer’s instructions. The isolated cells were then subjected to RNA extraction.

### Adeno-associated virus (AAV) preparation and stereotaxic injection

GLUT1 was encoded by gene *Slc2a1*. The GLUT1 knockdown AAV vector was designed to target at *Slc2a1* and constructed according to a previous protocol [23]. The target sequences used for knockdown are as follows: site1, 5′-CTCTGTCGGCCTCTTTGTTAA-3′; site2, 5′-ATGCGGGAGAAGAAGGTCACC-3′; site3, 5′-CTTCACTGTGGTGTCGCTGTT-3′. Besides, in order to initiate in microglia specifically, the promotor of the AAV vector was changed to CD68 promoter [24]. The AAV vector carrying green fluorescent protein (GFP) was used as control. The AAV vectors and viruses were prepared by Genchem Co., Ltd (Shanghai, China).

For viral injection, adolescent male C57BL/6J mice (4 weeks old) were anesthetized with 5% chloral hydrate (100 µL/10 g body weight) by intraperitoneal injection and placed on a stereotaxic apparatus. Small bilateral hole was drilled into the skull and the injection site was located into the hippocampal of right hemisphere using coordinate (-2.46 mm posterior of bregma, 1.7 mm lateral, and 2.0 mm depth). One microliter viruses (5.27 × 10^12^ vg/mL) were delivered via a Hamilton syringe at a rate of 0.1 µl per minute, and needles were kept still for an additional 1 min before withdrawing. The scalp was then sealed and injected mice were monitored as they recovered from anaesthesia. Arresting for at least 5 days after virus injection, mice were randomly assigned to CUMS procedure or control group.

### Cell culture and intervention

The mouse microglial BV2 cell line was purchased from BeNa Culture Collection (Beijing, China) and cultured in DMEM containing 10% Fetal Bovine Serum (FBS) and 100 U/mL penicillin and streptomycin (Solarbio, Beijing, China). Cells were grown in humidified incubator with 5% CO_2_ at 37°C.

GLUT1 expressing lentiviral vector was constructed by inserting the coding sequence (CDS) of mus *Slc2a1* gene (NM_011400) in lentiviral GV358 vector (Genechem, Shanghai, China) which contained GFP. Further, GLUT1 expressing lentivirus and stable transfectants of BV2 cells were established as described previously [25]. Briefly, HEK-293T cells were co-transfected with the lentiviral vector described above and packaging vectors psPAX2 and VSVG using lipo2000 (Invitrogen, California, USA). The lentivirus expressing GFP was used as control which purchased from Genechem Co., Ltd. (Shanghai, China). To construct of GLUT1 stable overexpression in microglial cells, BV2 cells were dissociated with 0.5% trypsin and seeded into six-well plates, following by infected with GLUT1 overexpression virus or the control virus. Then the stable infectants were screened by puromycin for 2 weeks.

Glucose (Sinopharm, Beijing, China) and GLUT1 antagonists, STF-31 and BAY-876 (MedChemExpress, New Jersey, USA), were added to the culture medium for intervention. Glucose was dissolved in 0.9% saline and the final concentration in the culture medium was 20 µmol/L. STF-31 and BAY-876 were dissolved in dimethyl sulfoxide (DMSO) (Sangon, Shanghai, China), and the final concentrations in the culture medium, chosen based on successful inhibition in previous publications, were 5 µmol/L and 50 nmol/L, respectively [26, 27]. An equal volume of saline or DMSO was used as control. After being incubated for 48 h, the cells were collected for further analysis.

### Cytoplasmic and nuclear protein extraction

Cytoplasmic and nuclear protein extraction was performed using a Nuclear and Cytoplasmic Protein Extraction Kit (Beyotime, Shanghai, China) following the manufacturer’s instructions. In brief, cells were harvested, and cell pellet was resuspended in an equal volume of regent A containing 1% PMSF. After a 10 min incubation on ice, cell lysates were mixed with 10 µL of regent B by vortexing and placed on ice for 1 min. Following centrifugation at 12,000 rpm, the supernatant containing the cytoplasmic protein fraction was collected, while the sediment was suspended in 50 µL of regent C. Samples were vortexed for 30 s every 2 min for a total of 15 cycles, after which the nuclear protein fraction was obtained by centrifugating at 12,000 rpm. The regent A, B, and C were provided by the Nuclear and Cytoplasmic Protein Extraction Kit mentioned above.

### Enzyme-linked immunosorbent assay (ELISA)

The brains or hippocampal tissues were removed from the stressed and the control mice followed by cutting into small pieces on ice. Then prechilled PBS was added and an electric homogenizer was used to homogenize sample fragment mixture. After centrifugation at 500 g for 5 min at 4 °C, the supernatant from the brain tissue was processed using Mouse Corticosterone ELISA Kit and NA/NE ELISA Kit (Sangon, Shanghai, China). Similarly, the supernatant from the hippocampus tissue was analyzed using Mouse IL-6, IL-1β and TNF-α ELISA kits (ABclonal, Wuhan, China). The collected cultured medium from BV2 cells was centrifugated at 900 g for 15 min at 4°C, and the supernatant was analyzed using Mouse IL-6, IL-1β, and TNF-α ELISA kits (ABclonal, Wuhan, China), following the manufacturer’s instructions.

### Quantitative real-time PCR (qRT-PCR)

Total RNA from tissues or cells was extracted using TRIzol reagent (Invitrogen, California, USA). The cDNA synthesis was performed using the ABScript II cDNA First-Strand Synthesis Kit (Abconal, Wuhan, China) according to the manufacturer’s instructions. Then qRT-PCR was conducted using a LightCycler 96 Realtime PCR System (Roche, Basel, Switzerland) with TB Green Premix Ex Taq kit (TaKaRa, Kyoto, Japan). The β-actin was selected as the endogenous control for the assay. The relative levels of the indicated genes were calculated by 2^−ΔΔCt^ method. The primers sequences used in this study are listed in Supplementary Table 1.

### Immunofluorescence (IF) staining

For immunohistofluorescence, the brains were separated and post-fixed in 4% paraformaldehyde (PFA) at 4°C overnight and immersed in 20% sucrose (4% PFA as solvent) followed by 30% sucrose (in 0.1 M PBS). The brain samples were cut into 20-µm-thick sections using a Leica CM1950 cryostat and subjected to immunohistofluorescence following routine protocols as described previously [28]. The antibodies used were provided in Supplementary Table 2. Fluorescence intensity was quantified using the Image J software.

For immunocytofluorescence, BV2 cells were cultured with indicated treatment for 48 h and subjected to IF staining based on a previously report [25]. Briefly, the cells were fixed in 4% PFA and permeabilized with 0.4% Triton X-100. After washing with TBS for 3 times, the cells were blocked with PBS containing 10% goat FBS (Solarbio, Beijing, China) and 1% BSA for 1 h and subsequently incubated with antibodies provided in Supplementary Table 2. The nucleus was counterstained with 4′,6-diamidino-2-phenylindole (DAPI) (Solarbio, Beijing, China). Images were captured using a confocal laser scanning microscope (Olympus, Tokyo, Japan).

### Glucose measurement of hippocampus

The hippocampal tissues were removed from both stressed and control mice brains and weighed. Then, 1 mL of distilled water was added per 0.1 g tissues, and homogenized using an electric homogenizer. The mixture was incubated in water bath at 95°C for 10 min followed by centrifugating at 8000 g for 10 min. The supernatant was collected and analyzed using Glucose Content Assay Kit (Sangon Biotech, Shanghai, China), following the manufacturer’s instructions.

### ^18^F-FDG PET scanning

Small-animal PET was performed according to a previous protocol [29, 30]. Briefly, mouse was anesthetized using 2% isoflurane and subsequently injected with ^18^F-FDG (180–230 µCi) via the tail vein. Immediately, the injected radiotracer was quantified using a dose calibrator (CAPINTEC, New Jersey, USA). The mouse, still under isoflurane anesthesia, was positioned prone within the field of view of a Micro-PET/CT scanner (PerkinElmer, Massachusetts, USA), and PET images were acquired 60 min after injection. The images were visualized and analyzed by VivoQuantTM software. The uptake of ^18^F-FDG was calculated as follows: normalized radioactivity = measured radioactivity in the PET image (kBq/mL)/injected radioactivity (kBq) in units of [%ID/mL].

### Flow cytometry analysis

Flow cytometry analysis was conducted as described previously [31]. BV2 cells were incubated with FITC-conjugated anti-CD86 antibody (Thermo, Massachusetts, USA). The cells were then fixed using IC Fixation Buffer (Thermo, Massachusetts, USA) and permeabilized with Permeabilization Buffer (Thermo, Massachusetts, USA). Subsequently, intracellular antigen staining was performed using PE-conjugated anti-CD206 antibody (Thermo, Massachusetts, USA). FITC-conjugated Rat IgG2a and PE-conjugated Rat IgG2b were employed as respective isotype controls. Cell populations were detected by using NovoCyte Flow Cytometer (Agilent Technologies, California, USA), and data were analyzed by using NovoExpress software.

### Western blotting

BV2 cells were lysed with Radio Immunoprecipitation Assay (RIPA) cell lysate (Solarbio, Beijing, China) supplemented with protease inhibitors, and subsequently centrifuged at 12,000 g for 15 min at 4°C to extract the supernatant. Next, loading buffer (Tiangen, Beijing, China) was added, and the samples were boiled for 5 min to denaturation. Electrophoresis was performed using a 10% SDS-PAGE gel, followed by transferring the proteins onto a polyvinylidene fluoride (PVDF) membrane using a wet transfer system (Bio-rad, California, USA). The membrane was blocked with 5% milk at room temperature for 1 h, washed 3 times with 1× TBST, and cut prior to hybridisation with antibodies. Primary antibodies were added separately and incubated overnight at 4°C. After washing 3 times with 1× TBST, the HRP labeled secondary antibodies were incubated for 2 h at room temperature. The membranes were prepared with ECL Western blotting substrate Kit (Termo Scientifc, Massachusetts, USA), followed by image acquisition using Image Quantlas 4000 (GE, NY, USA) and analysis using Image J software. The antibodies used in this study are listed in Supplementary Table 2.

For tissue samples, the hippocampus was cut into small pieces and homogenized using an electric homogenizer to create a mixture of sample fragment of RIPA lysate. The same procedure was then followed as for cell samples.

### Data analysis

Statistical analysis was processed with GraphPad Prism 8.0 software. The data was expressed as mean ± SD. The continuous variables were evaluated for normality before comparison for statistical differences. The significance of differences was assessed by two tail unpaired Student’s t-test or one-way or two-way analysis of variance (ANOVA) followed by Tukey’s multiple comparisons test. Pearson’s correlation analysis was performed to determine the correlation between two variables. The p-value less than 0.05 was considered to be significant.

## Results

### Chronic stress induces spatial learning and memory dysfunction in mice

**Fig. 1:**
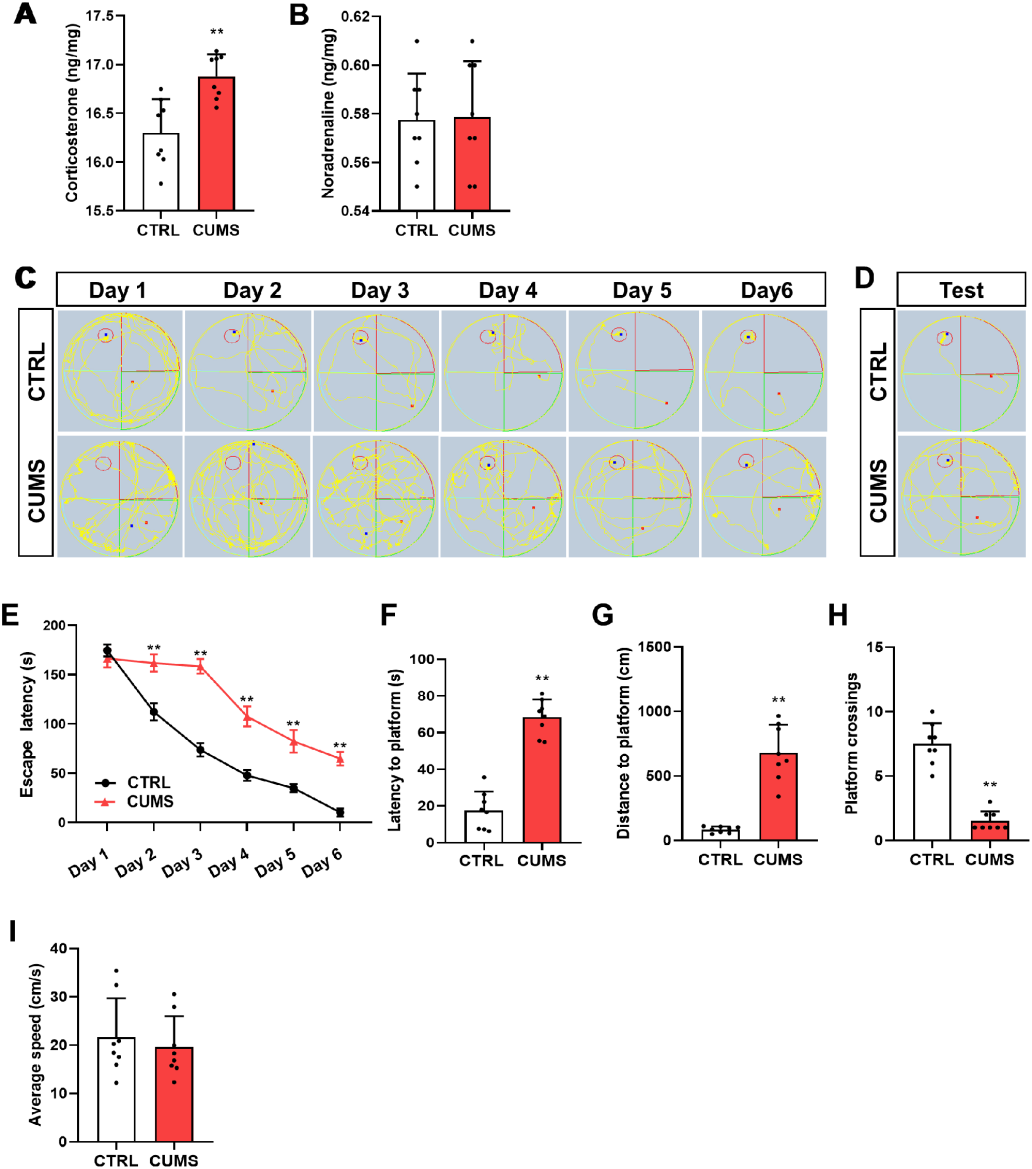
Chronic stress induces spatial learning and memory decline in mice. (A&B) Concentrations (ng/mg) of corticosterone (**A**) and norepinephrine (**B**) in the brain tissues of control and stressed mice at the end of the CUMS procedure (*n* = 8, Student’s t-test). (**C**&**D**) Representative track images of mice in the training trials (**C**) and the probe trial (**D**) of MWM. (**E**) Escape latency to the platform during the training trials in MWM (n = 8, Two-way ANOVA with Tukey’s post hoc test). (**F-I**) Latency to enter the platform (**F**), swimming distance of first time to enter the platform (**G**), platform crossings (**H**), and the average swimming speed (**I**) of mice in the probe trial of MWM (n = 8, Student’s t-test). **p < 0.01

Key hormones and cognitive behaviors were tested at the end of the CUMS procedure (8 w) to evaluate the effect of chronic stress on the brain. As shown in Fig. 1A and B, corticosterone levels were higher in the brains of stressed mice than in those of control mice, but noradrenaline levels were not, consistent with the knowledge that glucocorticoids are the predominant contributors to chronic stress-induced impairment [32]. More importantly, CUMS mice took longer than control mice to reach the platform in the training period of MWM task (Fig. 1C&E). Consistently, during the test period, CUMS mice took longer and swam further to reach the platform and demonstrated a decreased number of platform crossings but no obvious alteration in swimming speed (Fig. 1D and F-I), indicating chronic stress-induced spatial learning and memory deficits.

### Microglial proinflammatory activation is involved in the development of stress-induced spatial learning and memory dysfunction

**Fig. 2:**
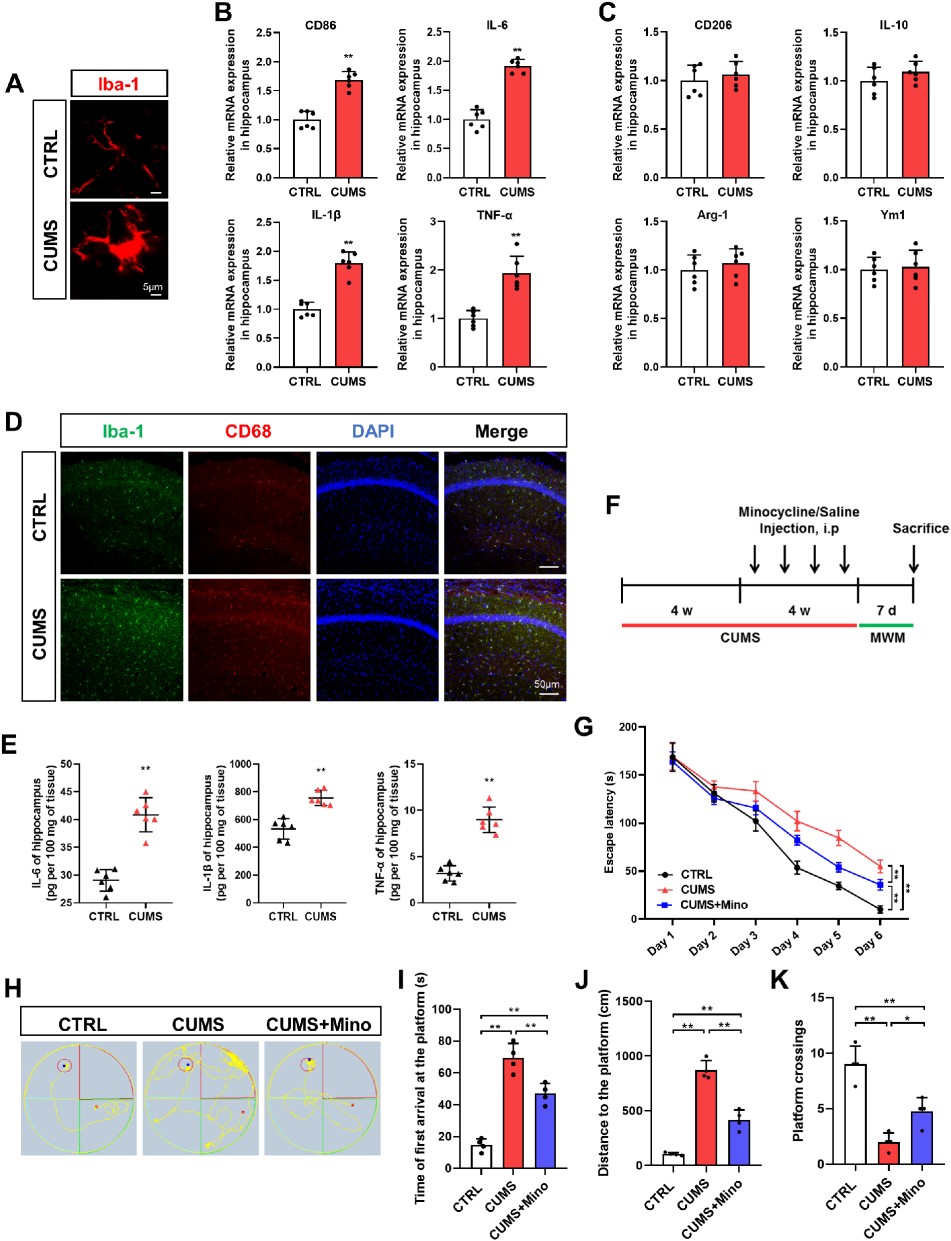
Microglia proinflammatory activation participates in stress-related learning and memory dysfunction. (**A**) Representative images of microglia in the hippocampus. Scale bar, 5 μm (n = 3). (**B**) qRT-PCR assays monitoring expression of proinflammatory phenotype markers, CD86, IL-6, IL-1β, and TNF-α in hippocampal samples from CTRL and CUMS mice (n = 6, Student’s t-test). (**C**) qRT-PCR assays monitoring expression of anti-inflammatory phenotype markers, CD206, IL-10, Arg-1, and Ym1 in hippocampal samples from CTRL and CUMS mice (n = 6, Student’s t-test). (**D**) Representative images of IF staining of hippocampal sections from CTRL and CUMS mice. Iba-1, green; CD68, red; DAPI, blue. Scale bar, 50 μm (n = 3). (**E**) Levels of IL-6, IL-1β, and TNF-α in hippocampus lysates from CTRL and CUMS mice as determined by ELISA (n = 6, Student’s t-test). (**F**) Schematic timeline of CUMS, Minocycline treatment, and MWM test. (**G**) Escape latency to the platform during the training trials in MWM of CTRL, CUMS, and CUMS + Minocyciline (Mino) mice (n = 4, Two-way ANOVA with Tukey’s post hoc test). (**H**) Representative track images of mice in the probe trial of MWM. (**I-K**) Latency to enter the platform (**I**), swimming distance of first time to enter the platform (**J**), and platform crossings (**K**) in the probe trial of MWM of CTRL, CUMS, and CUMS + Mino mice (n = 4, One-way ANOVA with Tukey’s post hoc test). *p < 0.05, **p < 0.01

As the hippocampus is the most important region in regulating spatial learning and memory, we investigated the influence of chronic stress on microglia in this brain region. Hippocampal tissues were collected at the end of the CUMS procedure (8 w). IF analysis with the microglia marker Iba-1 showed that CUMS promoted microglial activation, characterized a hypertrophic amoeboid shape and shortened, thickened processes (Fig. 2A). Because activated microglia can take on phenotypes which play opposite roles in neuroinflammation, we further examined the effect of chronic stress on microglial phenotypes. The expression levels of proinflammatory phenotype markers CD86, IL-6, IL-1β, and TNF-α, were elevated in CUMS mice (Fig. 2B), while no changes were observed in anti-inflammatory phenotype markers CD206, IL-10, Arg-1, and Ym1 (Fig. 2C). CD68, a key marker for proinflammatory-activated microglia, was further validated to be upregulated in CUMS mice by IF (Fig. 2D and Supplementary Fig. 1A). Additionally, a significant increase in the levels of inflammatory factors, including IL-6, IL-1β, and TNF-α, was observed in the hippocampus of CUMS mice by ELISA (Fig. 2E). The above results indicate microglial proinflammatory activation after chronic stress exposure. To further explore the role of microglia in stress-induced memory decline, minocycline was injected into CUMS mice to inhibit microglial activation (Fig. 2F). The CUMS-induced elevation of Iba-1 and CD68 were inhibited upon minocycline treatment (Supplementary Fig. 1B). Although differences remained between minocycline-treated stressed mice and control mice, the results of the MWM task showed that minocycline treatment alleviated the CUMS-induced prolonged learning time in the training period (Fig. 2G). Consistently, the swimming time and distance to reach the platform were shortened and the number of platform crossings was increased in CUMS mice treated with minocycline during the test period (Fig. 2H-K), suggesting improved memory function. Overall, these results indicate that chronic stress-induced microglial proinflammatory phenotype participates in learning and memory impairment.

### CUMS-induced hippocampal hyperglycemia is linked to microglial proinflammatory activation

**Fig. 3:**
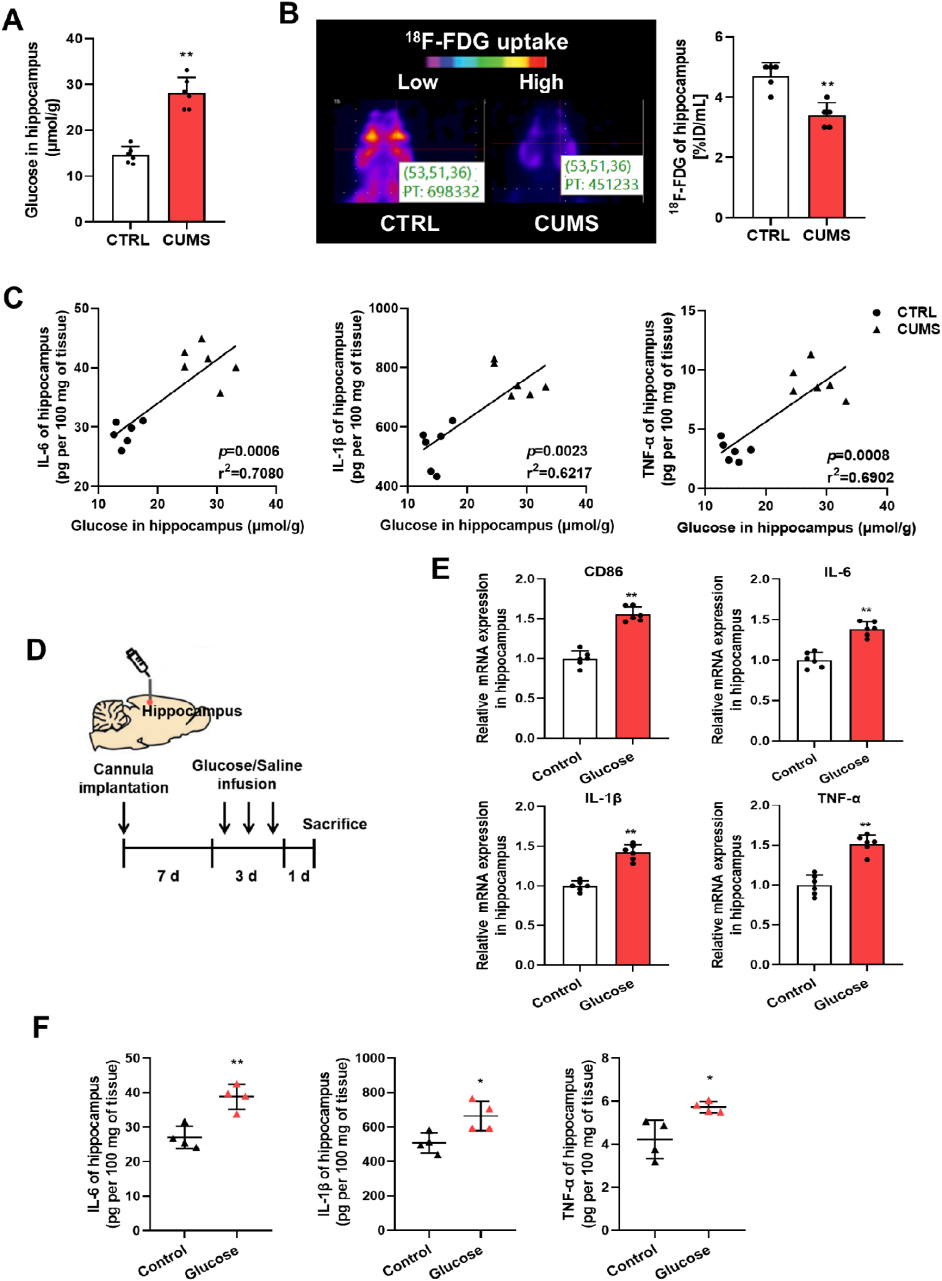
Hippocampal hyperglycemia contributes to CUMS-induced microglial proinflammatory activation. (**A**) Glucose levels in hippocampus lysates from CTRL and CUMS mice (n = 6, Student’s t-test). (**B**) Representative PET images of ^18^F-FDG uptake in the hippocampus in CTRL and CUMS mice (left) and the quantified result (right) (n = 5, Student’s t-test). (**C**) Correlation between glucose levels and IL-6, IL-1β, and TNF-α levels in the hippocampus (n = 12, Pearson’s correlation analysis). (**D**) Schematic of glucose infusion into the hippocampus and experimental timeline. Mice infused with saline were used as a control. (**E**) qRT-PCR assays monitoring the expression of proinflammatory phenotype markers, CD86, IL-6, IL-1β, and TNF-α in hippocampal samples from glucose-infused and control mice (n = 6, Student’s t-test). (**F**) Levels of IL-6, IL-1β, and TNF-α in hippocampus lysates from glucose-infused and control mice as determined by ELISA (n = 4, Student’s t-test). *p < 0.05, **p < 0.01

We measured hippocampal glucose levels to explore whether microglial activation is related to glucose metabolism in CUMS mice. As shown in Fig. 3A, the glucose level in hippocampal tissues was significantly upregulated in CUMS mice. Then, we measured ^18^F-FDG uptake by PET scanning, which was reduced in the hippocampus of CUMS mice versus controls (Fig. 3B). Intriguingly, the levels of inflammatory factors, IL-6, IL-1β, and TNF-α, correlated positively with the hippocampal glucose level (Fig. 3C). Thus, glucose was infused into hippocampal region of non-stressed mice, and the type of activated microglia was detected to elucidate the effect of hippocampal hyperglycemia on microglia activation (Fig. 3D). The results of qRT-PCR revealed an increase in the expression of proinflammatory phenotype markers (CD86, IL-6, IL-1β, and TNF-α) (Fig. 3E) but not of anti-inflammatory phenotype markers (CD206, IL-10, Arg-1, and Ym1) (Supplementary Fig. 2A) in the hippocampus after infusion with glucose. Moreover, glucose infusion increased IL-6, IL-1β, and TNF-α levels in hippocampal tissues (Fig. 3F). We then determined whether neuroinflammation could aggravate hippocampal hyperglycemia. LPS was injected into the hippocampus of normal mice, and the increases in inflammatory factors IL-6, IL-1β, and TNF-α were validated by ELISA (Supplementary Fig. 2B&C). However, the glucose level was not visibly changed in the hippocampus infused with LPS in comparison with that in the controls (Supplementary Fig. 2D). Thus, the data indicate that CUMS-induced hippocampal hyperglycemia promotes the activation of microglia toward the inflammatory phenotype.

### Glucose induces the proinflammatory phenotype in BV2 cells

**Fig. 4:**
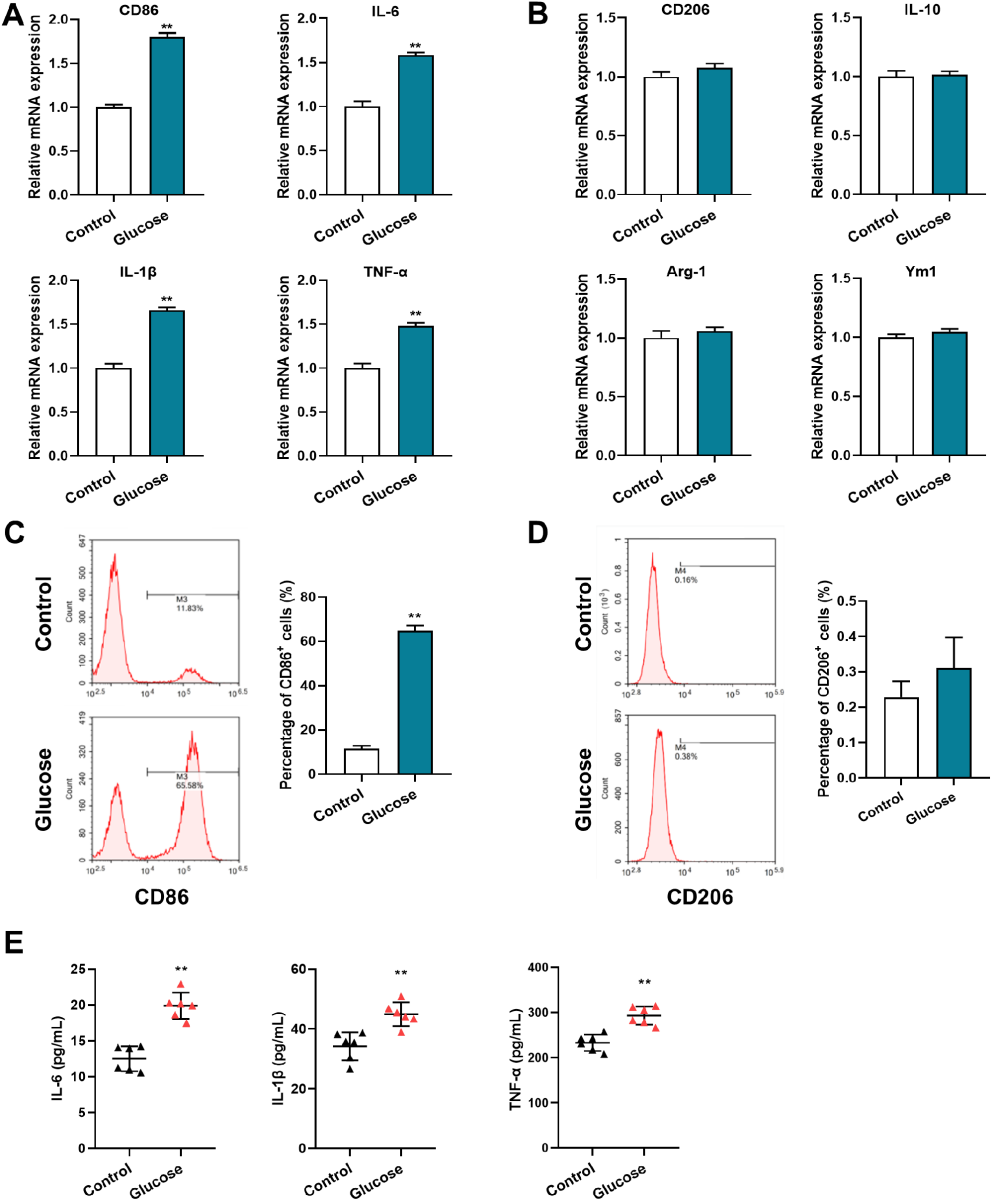
High glucose exposure promotes the proinflammatory phenotype of BV2 cells. (**A**) qRT-PCR assays monitoring the expression of proinflammatory phenotype markers, CD86, IL-6, IL-1β, and TNF-α in BV2 cells incubated with glucose (20 µmol/L) for 48 h. Control cells were incubated with saline (n = 6, Student’s t-test). (**B**) qRT-PCR assays monitoring expression of anti-inflammatory phenotype markers, CD206, IL-10, Arg-1, and Ym1 in glucose-treated BV2 cells and control cells (n = 6, Student’s t-test). (**C**) Flow cytometry analysis of CD86^+^ populations in glucose-treated BV2 cells and control cells. Representative images (left); quantified result (right, n = 5, Student’s t-test). (**D**) Flow cytometry analysis of CD206^+^ populations in glucose-treated BV2 cells and control cells. Representative images (left); quantified result (right, n = 5, Student’s t-test). (**E**) Secreted IL-6, IL-1β, and TNF-α proteins in the supernatant of glucose-treated BV2 cells and control cells as determined by ELISA (n = 6, Student’s t-test). **p < 0.01

The mouse microglial cell line BV2 was used to confirm the biological function of glucose. After incubation with glucose, the expressions levels of CD86, IL-6, IL-1β, and TNF-α in BV2 cells were increased, while those of CD206, IL-10, Arg-1, and Ym1 were unchanged (Fig. 4A&B). Flow cytometric analyses revealed an increased number of CD86^+^ but not CD206^+^ BV2 cells after glucose treatment in comparison with the control (Fig. 4C&D). Moreover, glucose promoted BV2 cell secretion of IL-6, IL-1β, and TNF-α (Fig. 4E), further indicating the proinflammatory phenotype transformation of BV2 cells after glucose exposure.

### GLUT1 expression is elevated in microglia isolated from the hippocampus of CUMS mice and high glucose-treated BV2 cells

**Fig. 5:**
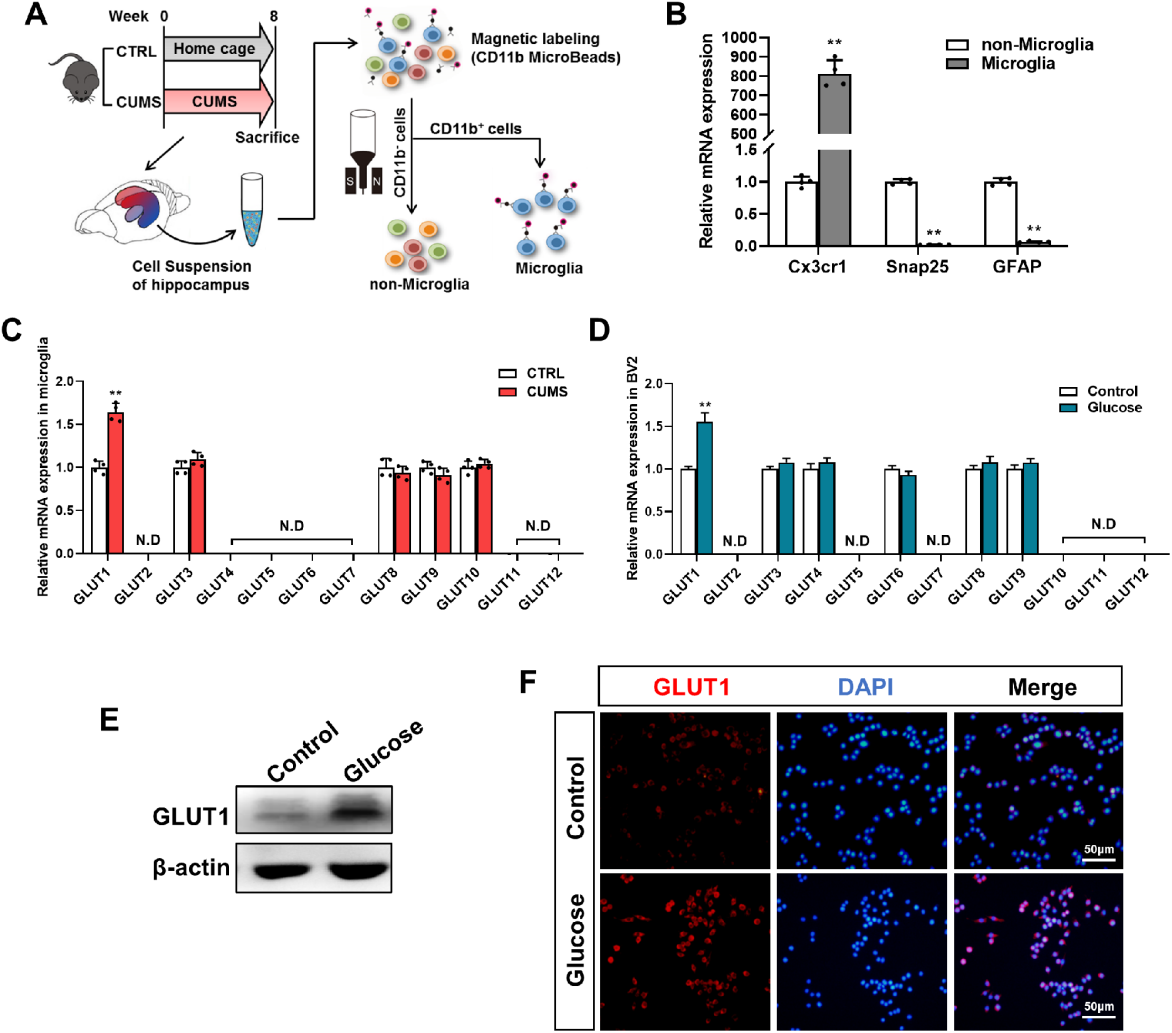
CUMS and glucose upregulate GLUT1 expression in microglia. (**A**) Schematic of microglia isolated from the hippocampus of CTRL and CUMS mice. (**B**) qRT-PCR assays validating the expression of biomarkers in isolated microglia versus non-microglia cells (n = 4, Student’s t-test). Cx3cr1, microglial biomarker; Snap25, neuronal biomarker; GFAP, astrocyte biomarker. (**C**) qRT-PCR assays monitoring the expression of GLUTs in derived microglia from CTRL and CUMS hippocampal tissues (n = 4, Student’s t-test). N.D, not detected. (**D**) qRT-PCR assays monitoring the expression of GLUTs in glucose-treated BV2 cells and control cells (n = 4, Student’s t-test). N.D, not detected. (**E**) Western blot of the expression of GLUT1 protein in glucose-treated BV2 cells and control cells. (**F**) Representative images of GLUT1 staining in glucose-treated BV2 cells and control cells. Scale bar, 50 μm. **p < 0.01

Microglia were magnetically separated from the hippocampus of CUMS mice and control mice to elucidate the mechanism underlying proinflammatory activation (Fig. 5A). As expected, microglial marker Cx3cr1 was highly expressed in the isolated cells, while neuronal marker Snap25 and astrocyte marker GFAP were almost undetectable (Fig. 5B), suggesting a good separation effect. GLUTs, the main transporters facilitating the entry of glucose into cells, are known to be associated with microglial activation [33]. Hence, we examined the expression of GLUT1–12 in the isolated microglia. The qRT-PCR analysis showed that only the expression of GLUT1 was elevated in microglia isolated from CUMS mice versus control microglia (Fig. 5C). Consistently, an increase in GLUT1 expression was observed in glucose-treated BV2 cells versus control cells by qRT-PCR (Fig. 5D), western blotting (Fig. 5E), and IF (Fig. 5F).

### GLUT1 is involved in the glucose-mediated proinflammatory phenotype in BV2 cells

**Fig. 6:**
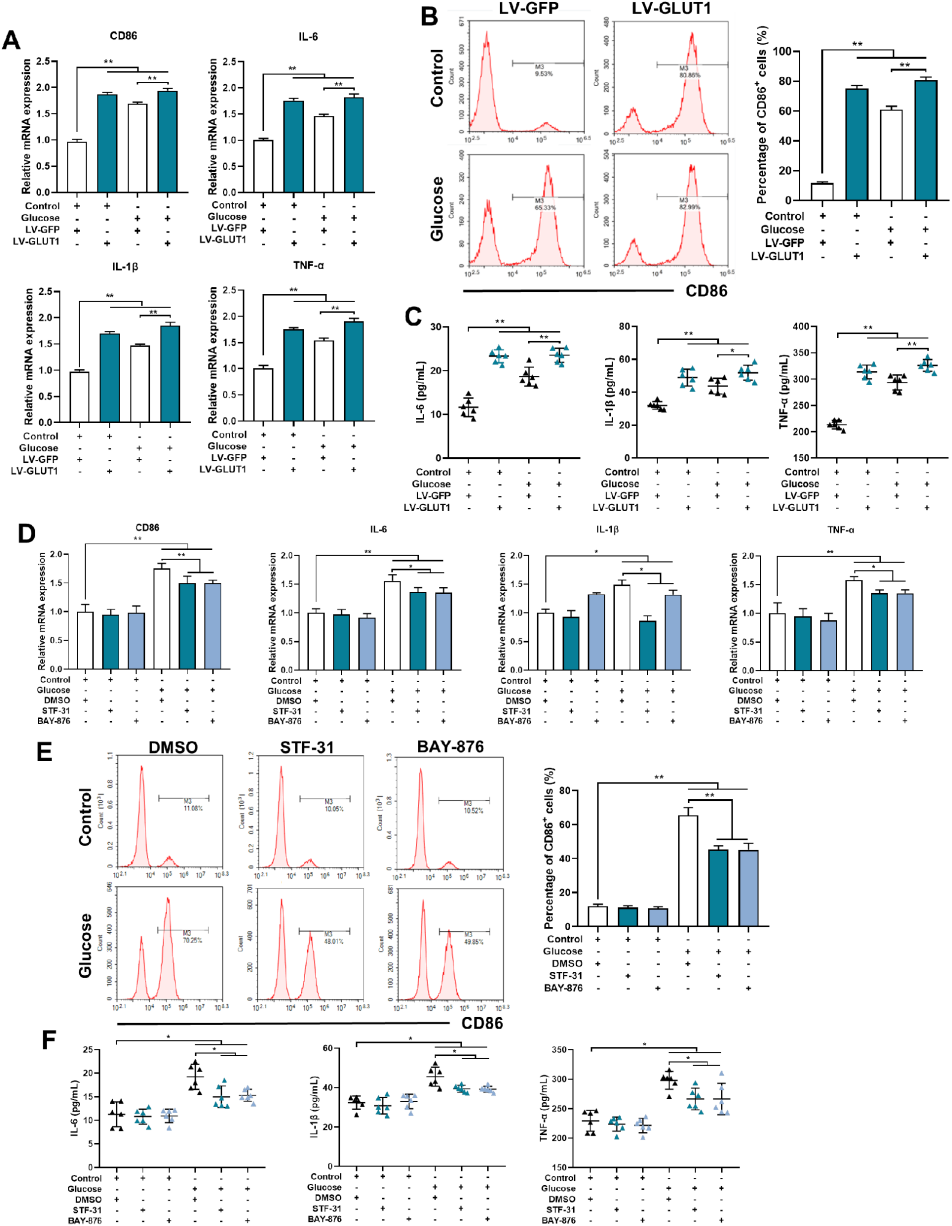
Glucose-mediates the proinflammatory activation of BV2 cells through GLUT1. (**A**) qRT-PCR assays monitoring expression of proinflammatory phenotype markers, CD86, IL-6, IL-1β, and TNF-α in LV-GFP- or LV-GLUT1-infected BV2 cells with or without glucose treatment (n = 6, One-way ANOVA with Tukey’s post hoc test). (**B**) Flow cytometry analysis of CD86^+^ populations in BV2-LV-GFP/LV-GLUT1 cells under control or glucose conditions. Representative images (left); quantified result (right, n = 5, One-way ANOVA with Tukey’s post hoc test). (**C**) Levels of secreted IL-6, IL-1β, and TNF-α proteins in the supernatant of BV2-LV-GFP/LV-GLUT1 cells with or without glucose treatment (n = 6, One-way ANOVA with Tukey’s post hoc test). (**D**) qRT-PCR assays evaluating the expression of proinflammatory phenotype markers, CD86, IL-6, IL-1β and TNF-α in BV2 cells exposed to GLUT1 inhibitors (STF-31, 5 µmol/L and BAY-876, 50 nmol/L) for 48 h with or without glucose treatment (n = 6, One-way ANOVA with Tukey’s post hoc test). (**E**) Flow cytometry analysis of CD86^+^ populations in BV2 cells exposed to STF-31 or BAY-876 under control or glucose conditions. Representative images (left); quantified result (right, n = 5, One-way ANOVA with Tukey’s post hoc test). (**F**) Levels of secreted IL-6, IL-1β and TNF-α proteins in the supernatant of BV2 cells exposed to STF-31 or BAY-876 with or without glucose treatment (n = 6, One-way ANOVA with Tukey’s post hoc test). *p < 0.05, **p < 0.01

The significance of GLUT1 in microglial activation was then explored by stably transfecting GLUT1 into microglial BV2 cells. BV2 cells expressing GFP were used as the control. The overexpression effect was confirmed by qRT-PCR and western blotting (Supplementary Fig. 3A&B). Compared with the control cells, with or without glucose exposure, GLUT1-overexpressing cells expressed higher mRNA levels of proinflammatory phenotype markers CD86, IL-6, IL-1β, and TNF-α (Fig. 6A). Additionally, the population of CD86^+^ cells was markedly increased in GLUT1-overexpressing BV2 cells versus GFP cells with saline or glucose treatment (Fig. 6B). GLUT1 overexpression promoted the secretion of IL-6, IL-1β, and TNF-α and facilitated the induction of these factors by glucose (Fig. 6C). Nevertheless, GLUT1 did not affect the expression of most anti-inflammatory phenotype markers, such as CD206, IL-10, and Ym1; Arg-1 was slightly upregulated by GLUT1 overexpression (Supplementary Fig. 3C). The population of CD206^+^ cells did not change among GLUT1-overexpressing BV2 cells versus GFP cells regardless of glucose exposure (Supplementary Fig. 3D). Additionally, STF-31 and BAY-87, antagonists of GLUT1, reduced glucose-induced CD86, IL-6, IL-1β, and TNF-α expression, the population of CD86^+^ cells, and the secretion of IL-6, IL-1β, and TNF-α (Fig. 6D-F). The expression levels of anti-inflammatory phenotype markers and the population of CD206^+^ cells were not affected by GLUT1 antagonists (Supplementary Fig. 3E&F). Overall, these data indicate a facilitating role of GLUT1 in glucose-induced microglial proinflammatory activation.

### GLUT1-specific knockdown in hippocampal microglia alleviates microglia proinflammatory activation and learning and memory impairment in CUMS mice

**Fig. 7:**
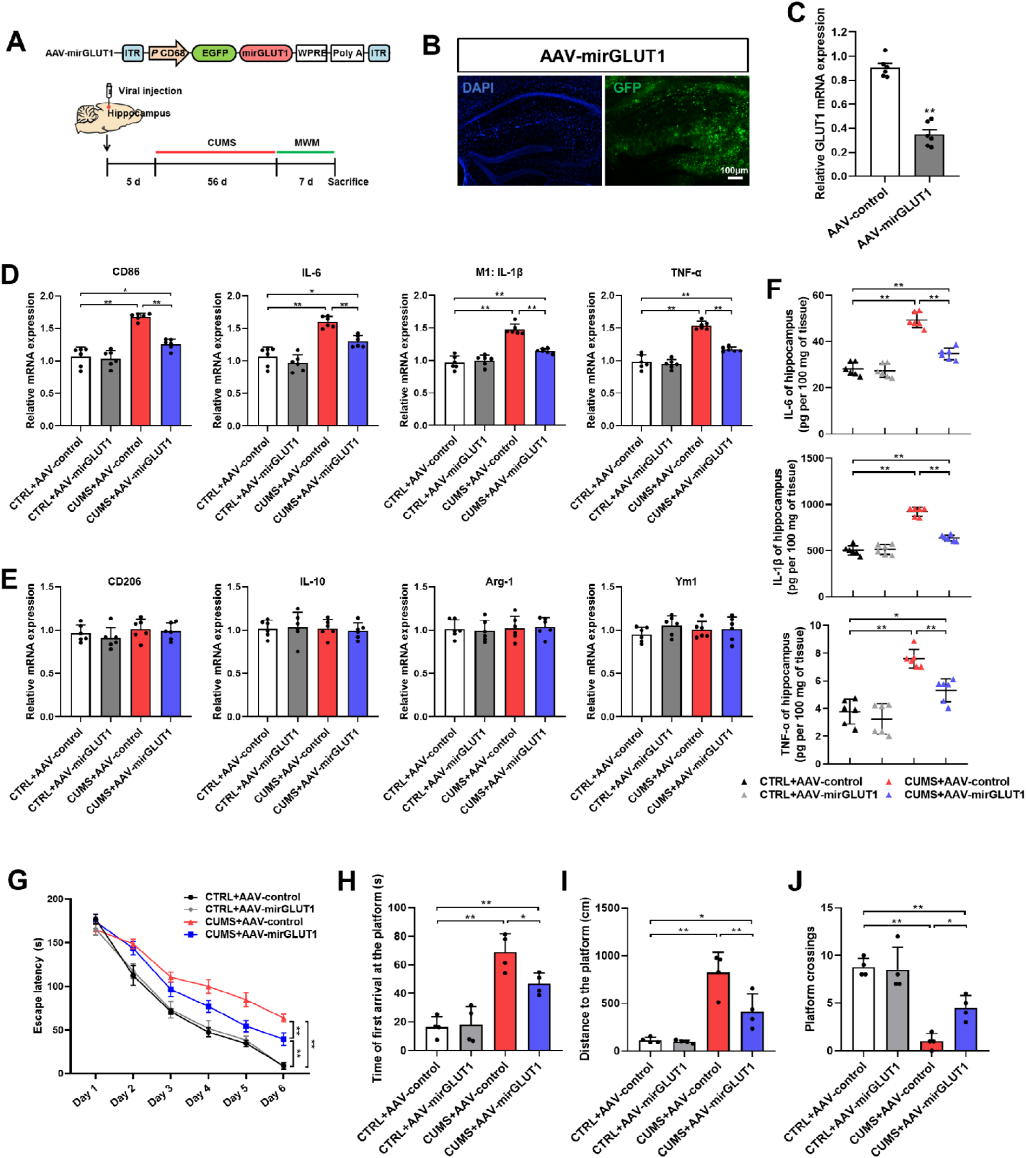
GLUT1-specific knockdown in hippocampal microglia alleviates microglial proinflammatory state and CUMS-related spatial learning and memory impairment. (**A**) Schematics of the AAV construct expressing GLUT1-targeted microRNAs specifically in microglia (AAV-mirGLUT1) (upper). AAV-mirGLUT1 or AAV-control was injected into the mouse hippocampus, and the experimental timeline is shown (lower). (**B**) Representative fluorescence image of GFP in the mouse hippocampus after AAV-mirGLUT1 infection. Scale bar, 100 μm. (**C**) qRT-PCR assay monitoring the expression of GLUT1 in the mouse hippocampus injected with AAV-mirGLUT1 or AAV-control (n = 6, Student’s t-test). (D&E) qRT-PCR assays monitoring expression levels of proinflammatory phenotype markers (**D**) and anti-inflammatory phenotype markers (**E**) in hippocampal samples from CTRL and CUMS mice infected with AAV-mirGLUT1 or AAV-control (n = 6, One-way ANOVA with Tukey’s post hoc test). (**F**) Levels of IL-6, IL-1β, and TNF-α in hippocampus lysates from CTRL and CUMS mice infected with AAV-mirGLUT1 or AAV-control as determined by ELISA (n = 6, One-way ANOVA with Tukey’s post hoc test). (**G**) Escape latency to the platform during the training trials in the MWM test of CTRL and CUMS mice injected with AAV-mirGLUT1 or AAV-control (n = 4, Two-way ANOVA with Tukey’s post hoc test). (**H-J**) Latency to enter the platform (H), swimming distance of first time to enter the platform (**I**), and platform crossings (**J**) in the probe trial of the MWM test of CTRL and CUMS mice after AAV-mirGLUT1 or AAV-control infection (n = 4, One-way ANOVA with Tukey’s post hoc test). *p < 0.05, **p < 0.01

An AAV specifically inhibiting GLUT1 expression in microglia (AAV-mirGLUT1) was constructed and microinjected into the hippocampus (Fig. 7A). The successful infection of hippocampal tissue was validated by GFP expression (Fig. 7B). The knockdown of GLUT1 in the hippocampus after AAV-mirGLUT1 infection was confirmed by qRT-PCR (Fig. 7C). Next, the influence of GLUT1 depletion on microglial activation was examined. IF staining results showed that the levels of CD68 were decreased in the hippocampus of AAV-mirGLUT1 injected mice (Supplementary Fig. 4A&B). Additionally, GLUT1 knockdown inhibited the expression of CD86, IL-6, IL-1β, and TNF-α induced by CUMS in the hippocampal tissues, with no change in CD206, IL-10, Arg-1, and Ym1 (Fig. 7D&E). Consistently, decreased levels of IL-6, IL-1β, and TNF-α were found in the hippocampus of CUMS mice infected with AAV-mirGLUT1 versus control AAV (Fig. 7F). Furthermore, the MWM test was used to assess spatial learning and memory abilities. AAV-mirGLUT1 had no effect on the learning and memory abilities of normal mice (Fig. 7G-J). Nevertheless, a shorter latency to reach the platform during the training trial was observed in CUMS mice injected with AAV-mirGLUT1 versus those injected with control AAV (Fig. 7G). Moreover, repressing the expression of GLUT1 in the hippocampal microglia of CUMS mice resulted in a shorter time and swimming distance before the first entry into the target and an increased number of platform crossings (Fig. 7H-J), indicating better memory retention. Collectively, these results demonstrate that GLUT1-specific knockdown in hippocampal microglia inhibits its proinflammatory activation and improves spatial learning and memory abilities in CUMS mice.

### GLUT1 modulates NF-κB signaling in microglia

**Fig. 8:**
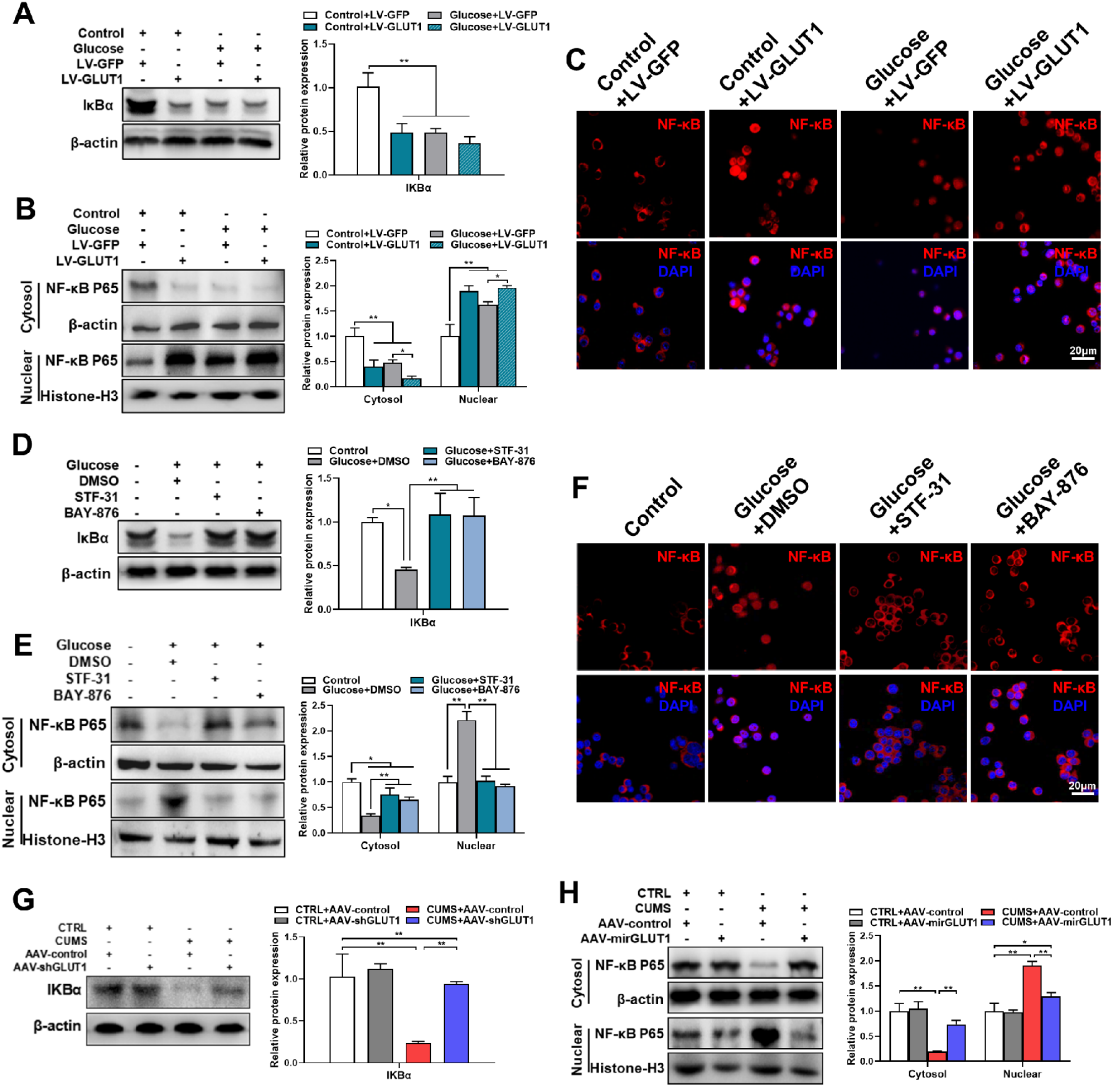
Glucose and CUMS inhibited IκBα expression and facilitated NF-κB p65 nuclear translocation via GLUT1. (**A**) Levels of IκBα proteins in BV2-LV-GFP/LV-GLUT1 cells treated with or without glucose were determined by western blotting, and the quantified result is shown (n = 3, One-way ANOVA with Tukey’s post hoc test). (**B**) NF-κB p65 in the cytosolic/nuclear fractions of BV2-LV-GFP/LV-GLUT1 cells treated with or without glucose was determined by western blotting. The quantified result is shown (n = 3, One-way ANOVA with Tukey’s post hoc test). (**C**) NF-κB nuclear translocation in BV2-LV-GFP/LV-GLUT1 cells under control or glucose conditions was analyzed by IF staining. Representative images are shown. Scale bar, 20 μm. (**D**) Levels of IκBα in BV2 cells exposed to STF-31 or BAY-876 upon glucose treatment were determined by western blotting. The quantified result is shown (n = 3, One-way ANOVA with Tukey’s post hoc test). (**E**) NF-κB p65 in the cytosolic/nuclear fractions of BV2 cells exposed to STF-31 or BAY-876 upon glucose treatment was determined by western blotting. The quantified result is shown (n = 3, One-way ANOVA with Tukey’s post hoc test). (**F**) NF-κB nuclear translocation in BV2 cells exposed to STF-31 or BAY-876 under glucose incubation was analyzed by IF staining. Representative images are shown. Scale bar, 20 μm. (**G&H**) Levels of IκBα (**G**), and NF-κB p65 in cell cytosolic/nuclear fractions (**H**) in hippocampus lysates from CTRL and CUMS mice with AAV-mirGLUT1 or AAV-control injection were determined by western blotting. The quantified result is shown (n = 3, One-way ANOVA with Tukey’s post hoc test). *p < 0.05, **p < 0.01

NF-κB signaling is one of the most important pathways mediating microglial activation [34]. Hence, we investigated the influence of glucose on NF-κB signaling. Western blotting demonstrated that the level of IκBα protein, a negative regulator of NF-κB signaling, was downregulated in glucose-treated BV2 cells versus control cells (Supplementary Fig. 5A). Additionally, cytosolic NF-κB p65 was decreased and nuclear NF-κB p65 was increased after glucose incubation (Supplementary Fig. 5B). IF validated the enhanced nuclear translocation of NF-κB p65 with glucose exposure (Supplementary Fig. 5C). We next explored whether glucose modulated NF-κB signaling *via* GLUT1. The results revealed that GLUT1 overexpression inhibited the expression of IκBα and promoted NF-κB p65 nuclear translocation (Fig. 8A-C). In addition, GLUT1 antagonism inhibited glucose-induced IκBα suppression and NF-κB p65 nuclear translocation in BV2 cells (Fig. 8D-F). Furthermore, compared to the control group, CUMS mice showed reduced expression of IκBα protein and increased NF-κB p65 nuclear translocation in hippocampal tissues, which were ameliorated by the knockdown of GLUT1 specifically in hippocampal microglia (Fig. 8G&H). Thus, these results indicate that glucose and CUMS promotes the activation of NF-κB signaling in microglia *via* GLUT1.

## Discussion

The pathogenesis of neuroinflammation in various neurological disorders has attracted significant attention in recent years. Numerous clinical and preclinical studies have confirmed a strong correlation between neuroinflammation and cognitive disorders, including Alzheimer’s disease and stress-related cognitive impairment [35, 36]. Therapies targeting neuroinflammation have also shown promising results in alleviating cognitive dysfunctions [37, 38]. Microglia are emerging as critical key regulators of the inflammatory response process in the central nervous system; overactivation of these cells might impair cognition through the excessive release of inflammatory cytokines [9]. Microglia rapidly respond to changes in their microenvironment during stress, which might lead to a maladaptive or proinflammatory state in conditions of prolonged stress exposure [8]. Indeed, numerous studies have demonstrated that chronic stress can selectively increase the number of activated microglia and induce their inflammatory polarization in stress-associated brain regions [39]. However, the underlying mechanism remains incompletely understood.

Here, we have identified hippocampal hyperglycemia as a distinct alteration induced by chronic stress that correlates significantly with microglial inflammatory activation. Many studies have suggested that hyperglycemia in diabetes can induce dramatic inflammatory reactions in retinal and myocardial cells [40, 41]. Hyperglycemia is also very common in stress-related diseases. Glucocorticoid (GC), the most famous stress hormone, was named for its significant role in regulating glucose metabolism and can inhibit glucose consumption, promote gluconeogenesis, and cause hyperglycemia. Our findings and those reported in other studies suggest that changes in glucose metabolism are involved in the regulation of hippocampal neuroinflammation, potentially linking chronic stress and proinflammatory microglial activation. There is a clear linkage and complex crosstalk among inflammation, glucose metabolism, and stress. Systemic low-grade inflammation is a common feature of chronic metabolic disorders and stress-related diseases [42]. Metabolic disorders are also present in stressed organisms and can interact with inflammatory responses [43]. For example, it has been found that metabolic disorders can drive neuroinflammation and facilitate the development of neurodegenerative diseases [44]. Terms such as metaflammation and immunometabolism have been coined to describe the close relationship between inflammation and metabolism [45, 46]. Our data also demonstrate that both intracerebral administration of glucose in vivo and high glucose exposure in vitro are sufficient to lead to microglial proinflammatory phenotype, confirming the possible central role of stress-induced hippocampal hyperglycemia in inducing microglial inflammation and subsequent spatial learning and memory impairment. In accordance with our results, other studies also suggested that high glucose or hyperglycemia could induce the proinflammatory polarization of hepatic macrophages or cerebral microglia and subsequently aggravate inflammation in the liver and brain [16, 47]. Moreover, other studies have indicated that inflammation can also induce abnormal glucose metabolism and lead to hyperglycemia [48]. To test the possible effects of inflammation on glucose metabolism, we injected LPS into the hippocampus of normal mice and found that although dramatic inflammation appeared after LPS injection, the glucose level in the hippocampus was not changed; this indicated that the alteration in glucose metabolism was likely to occur prior to inflammation in the hippocampus of stress mice.

Why cerebral hyperglycemia occurs during chronic stress remains unclear and has been thought to be associated with GC-induced hyperphagia, especially in the late period of chronic stress with the subsiding glucose consumption and energetic demand [49]. However, our ^18^F-FDG data revealed that hyperglycemia is accompanied by reduced glucose uptake in the stress-exposed hippocampus, reflecting not only an imbalance between glucose supply and demand in the brain but also abnormal glucose metabolism. It is unusual to observe conflicting results between higher hippocampal glucose levels and lower hippocampal ^18^F-FDG uptake in our stressed animal model. However, we hypothesize that this could be explained by competition between endogenous glucose and available glucose uptake sites. According to previous investigations, similar contradictions have been found in humans undergoing acute glucose loading and mice with hyperglycemia induced by chronic social stress [16, 50]. As previously reported, peripheral hyperglycemia under stress or declining neurocellular glucose consumption potentially explains the hippocampal hyperglycemia [16]. Glucose is transported into cells via GLUTs for utilization. However, our data indicate an increase, rather than a decrease, in GLUT1 expression in microglia isolated from CUMS animals or exposed to high glucose incubation. The results suggest that the relatively smaller proportion of microglia in nervous tissue may not be the main contributors to overall hippocampal glucose levels.

Although the expression of GLUTs is usually increased to facilitate glucose transport in response to low glucose levels, some studies have found that exposure to high glucose leads to upregulation of GLUT1 expression in macrophages [18], mesangial cells [51], and pancreatic cancer cells [52]. Furthermore, elevated GLUT1 expression in the brain has been observed in individuals experiencing acute hyperglycemia induced by oral glucose loading [53]. In this study, we found that both stress and high glucose exposure upregulated GLUT1 expression in microglia. When activated to a proinflammatory phenotype, microglia undergo cytoskeletal changes, functional remodeling, and cytokine synthesis, all of which require a vast amount of energy [12, 54]. The increased GLUT1 level triggered by chronic stress may be associated with the high energy demand of activated microglia, which merits further exploration.

Adolescence is a crucial developmental period marked by significant changes in behavior and cognition, which are believed to align with the maturation of brain circuits essential for learning and memory, particularly in the hippocampus. This stage is also characterized by the presence of various psychosocial and physical stressors, making it a pivotal time for the emergence of psychiatric disorders such as stress disorders, mood disorders, obsessive-compulsive disorder, and schizophrenia [55]. Accumulating evidence indicates that chronic stress, including CUMS, social isolation, and restraint stress, can reduce hippocampal neurogenesis in adolescents, leading to impaired hippocampal-dependent learning and memory, as well as depressive-like behaviors that extend into adulthood [56, 57]. Therefore, it is crucial to develop therapeutic strategies targeting neurogenic mechanisms during this critical developmental phase in individuals exposed to stress. The findings of this study support the idea that manipulating GLUT1 levels in adolescent mice can significantly impact microglial proinflammatory activation and mitigate stress-induced cognitive impairment in adulthood, offering a potential target for preventing cognitive deficits following stress exposure.

Our finding suggests that the upregulation of GLUT1 may play a causal role in the inflammatory polarization of microglia under stress conditions. Similar observations have been reported in the context of vascular injury and heart disease, where upregulation of GLUT1 has been shown to promote inflammation in smooth muscle cells, cardiac myocytes, and endothelial cells. Consequently, targeting GLUT1 using inhibitors may be a promising pharmacological approach to restrict pathological inflammation [58, 59]. NF-κB signaling, a critical mediator of inflammation, was identified as a switch for the proinflammatory activation of microglia. Our findings confirmed that both stress and GLUT1 could activate NF-κB signaling by suppressing the expression of IκBα and facilitating the nuclear translocation of p65. This observation is consistent with the results of another study, where it was found that GLUT1 inhibition reduced NF-κB pathway activation [60, 61]. The detailed mechanism by which GLUT1 affects inflammation remains to be elucidated. Considering that GLUT1 functions as a key glucose transporter and upregulation acts as a marker for aerobic glycolysis in metabolically active cells [62], we speculate that the regulation of GLUT1 in microglial inflammation might be associated with metabolic reprogramming. This hypothesis is corroborated by previous findings that the overexpression or increased expression of GLUT1 could decrease macrophage oxygen consumption, increase cellular glucose uptake, and enhance glycolytic capacity, leading to excessive activation and increased secretion of inflammatory mediators [63]. Conversely, GLUT1 antagonists have been found to decrease glycolytic levels in microglia and inhibit their inflammatory activation [33]. Combining these previous findings with ours indicates that GLUT1 may be an important target in the stress-induced inflammatory polarization of hippocampal microglia.

Chronic stress, in addition to cognitive impairment, is recognized as a significant risk factor for mood disorders, particularly depressive disorder. The CUMS procedure is commonly utilized as an animal model of depression [20, 64, 65]. However, the pathogenesis of stress-related depression is remains incompletely understood. Disturbances in glucose metabolism disturbance and neuroinflammation are two important hypothesized causes of depression [66, 67]. Clinical studies have highlighted the frequent co-occurrence of depression and diabetes, with depression being twice as prevalent in individuals with diabetes compared to the general population [68]. Elevated blood glucose levels have also been observed in animal models of stress-induced depression [69, 70], although research on glucose levels in specific brain regions remains limited. The relationship between inflammation and depression has garnered significant attention, with cumulative evidence suggesting that inflammatory processes play a crucial role in the initiation and development of depression. Our team’s previous research demonstrated that injection of TNF-α into the hippocampus evoked depression-like behaviors [28]. Besides, the facilitative role of brain IL-1β in the pathogenesis of depression has also been revealed [71, 72], confirming the contribution of central inflammatory cytokines in depression. In the present study, we observed that CUMS led to pronounced hippocampal hyperglycemia, contributing to neuroinflammation and spatial memory dysfunction through GLUT1-mediated microglial proinflammation activation. However, as this study focused on stress-related cognitive impairment, the interplay between hippocampal hyperglycemia, brain inflammatory cytokines and depressive behaviors was not explored. Given the crucial role of GLUT1 in glucose transport and neuroinflammation elucidated in this study, it is plausible that GLUT1 is implicated in the pathogenesis of stress-induced depression, warranting further comprehensive exploration.

This study does have some limitations. First, the underlying cause of the increase in microglial GLUT1 expression during chronic stress remains unknown. Future studies are needed to investigate whether GLUT1 upregulation is a proactive response by the body to stress or a reactive response to stress-induced hyperglycemia in the hippocampus. Second, the precise mechanism through which GLUT1 activates the NF-κB signaling pathway requires further elucidation and validation.

## Conclusions

**Fig. 9:**
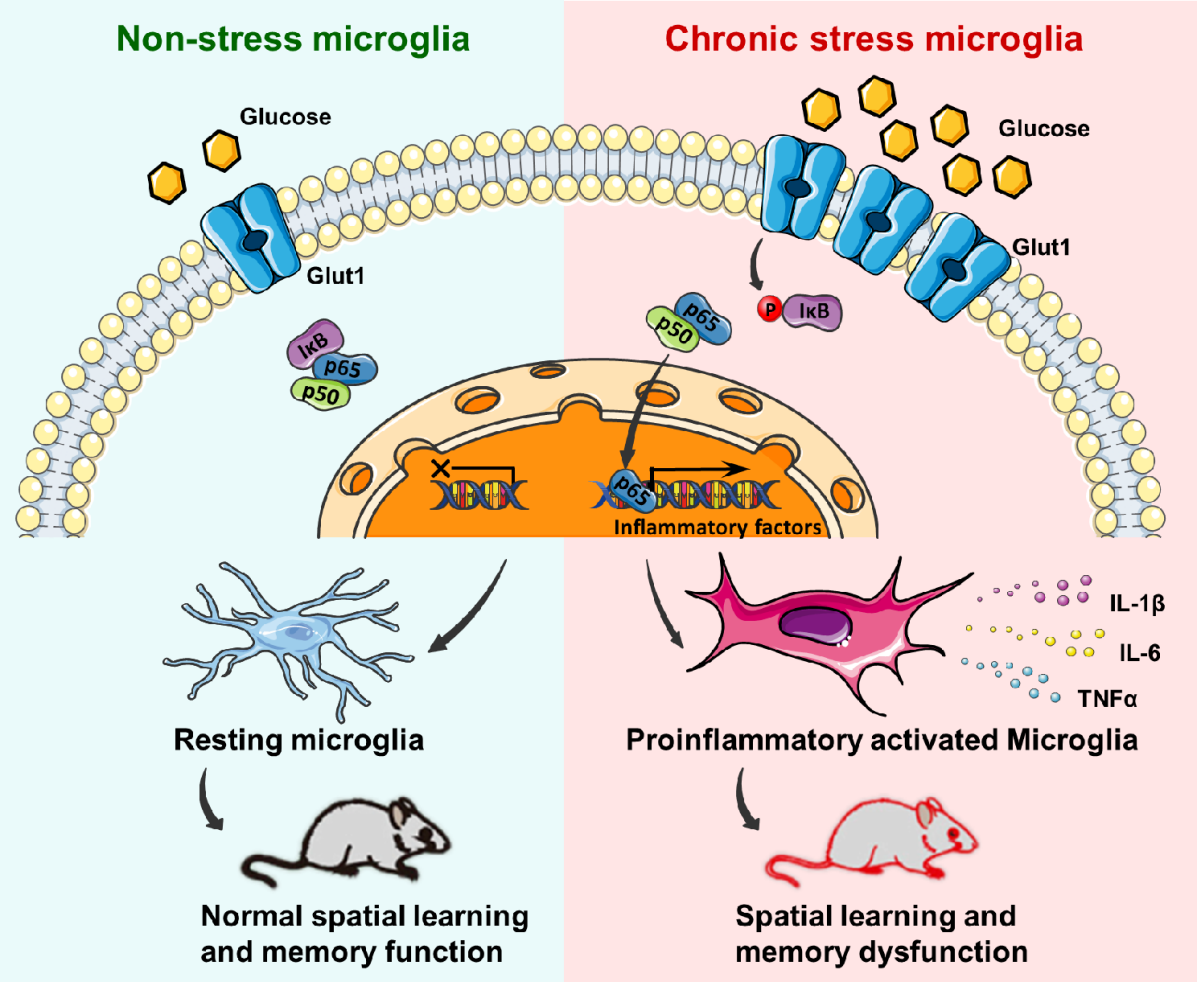
A schematic model of the glucose-induced proinflammatory activation of microglia in chronic stress-related spatial cognitive dysfunction

Overall, our findings demonstrate that disordered glucose metabolism could be induced by chronic stress, which plays a key role in the proinflammatory activation of hippocampal microglia through GLUT1 and NF-κB signals (Fig. 9). This remarkable crosstalk between metabolism and inflammation in stress disorders provides a possible therapeutic target against stress-related cognitive dysfunction. It would be a possible way to use nutriologial methods to improve cerebral glucose metabolism, helping to control neuroinflammation in the hippocampus and to alleviate cognitive injury during stress.

### Electronic supplementary material

Below is the link to the electronic supplementary material.

Supplementary Material 1

## Data Availability

All data generated or analyzed during this study are included in this published article and its supplementary information files.

## Abbreviations

IL-6: interleukin-6
IL-1β: interleukin-1β
TNF-α: tumor-necrosis factor-α
GLUTs: glucose transporters
CUMS: chronic unpredictable mild stress
MWM: Morris water maze
AAV: adeno-associated virus
GFP: green fluorescent protein
FBS: fetal bovine serum
CDS: coding sequence
DMSO: dimethyl sulfoxide
ELISA: enzyme-linked immunosorbent assay
qRT-PCR: quantitative real-time PCR
IF: immunofluorescence
PFA: paraformaldehyde
DAPI: 4′,6-diamidino-2-phenylindole
RIPA: radio immunoprecipitation assay
PVDF: polyvinylidene fluoride
ANOVA: analysis of variance
GC: glucocorticoid

## References

[1] Greenberg MS, Tanev K, Marin MF, Pitman RK (2014). Stress, PTSD, and dementia. Alzheimers Dement.

[2] Lutz MW, Luo S, Williamson DE, Chiba-Falek O (2020). Shared genetic etiology underlying late-onset Alzheimer’s disease and posttraumatic stress syndrome. Alzheimers Dement.

[3] Polis B, Karasik D, Samson AO (2021). Alzheimer’s disease as a chronic maladaptive polyamine stress response. Aging.

[4] McEwen BS, Bowles NP, Gray JD, Hill MN, Hunter RG, Karatsoreos IN, Nasca C (2015). Mechanisms of stress in the brain. Nat Neurosci.

[5] Shen J, Li Y, Qu C, Xu L, Sun H, Zhang J (2019). The enriched environment ameliorates chronic unpredictable mild stress-induced depressive-like behaviors and cognitive impairment by activating the SIRT1/miR-134 signaling pathway in hippocampus. J Affect Disord.

[6] Wang SD, Wang X, Zhao Y, Xue BH, Wang XT, Chen YX, Zhang ZQ, Tian YR, Xie F, Qian LJ (2022). Homocysteine-Induced disturbances in DNA methylation contribute to development of stress-Associated Cognitive decline in rats. Neurosci Bull.

[7] Xie F, Zhao Y, Ma J, Gong JB, Wang SD, Zhang L, Gao XJ, Qian LJ (2016). The involvement of homocysteine in stress-induced Abeta precursor protein misprocessing and related cognitive decline in rats. Cell Stress Chaperones.

[8] Fonken LK, Frank MG, Gaudet AD, Maier SF (2018). Stress and aging act through common mechanisms to elicit neuroinflammatory priming. Brain Behav Immun.

[9] Woodburn SC, Bollinger JL, Wohleb ES (2021). The semantics of microglia activation: neuroinflammation, homeostasis, and stress. J Neuroinflammation.

[10] Subhramanyam CS, Wang C, Hu Q, Dheen ST (2019). Microglia-mediated neuroinflammation in neurodegenerative diseases. Semin Cell Dev Biol.

[11] Nimmerjahn A, Kirchhoff F, Helmchen F (2005). Resting microglial cells are highly dynamic surveillants of brain parenchyma in vivo. Science.

[12] Yang S, Qin C, Hu ZW, Zhou LQ, Yu HH, Chen M, Bosco DB, Wang W, Wu LJ, Tian DS (2021). Microglia reprogram metabolic profiles for phenotype and function changes in central nervous system. Neurobiol Dis.

[13] Hinwood M, Morandini J, Day TA, Walker FR (2012). Evidence that microglia mediate the neurobiological effects of chronic psychological stress on the medial prefrontal cortex. Cereb Cortex.

[14] Messier C (2004). Glucose improvement of memory: a review. Eur J Pharmacol.

[15] van der Zwaluw NL, van de Rest O, Kessels RP, de Groot LC (2015). Effects of glucose load on cognitive functions in elderly people. Nutr Rev.

[16] van der Kooij MA, Jene T, Treccani G, Miederer I, Hasch A, Voelxen N, Walenta S, Muller MB (2018). Chronic social stress-induced hyperglycemia in mice couples individual stress susceptibility to impaired spatial memory. Proc Natl Acad Sci U S A.

[17] Nguyen YTK, Ha HTT, Nguyen TH, Nguyen LN (2021). The role of SLC transporters for brain health and disease. Cell Mol Life Sci.

[18] Wang Q, Nie L, Zhao P, Zhou X, Ding Y, Chen Q, Wang Q (2021). Diabetes fuels periodontal lesions via GLUT1-driven macrophage inflammaging. Int J Oral Sci.

[19] Qiao H, Li MX, Xu C, Chen HB, An SC, Ma XM. Dendritic Spines in Depression: What We Learned from Animal Models. Neural Plast 2016, 2016:8056370. 10.1155/2016/8056370 PMC4736982 26881133

[20] Leng L, Zhuang K, Liu Z, Huang C, Gao Y, Chen G, Lin H, Hu Y, Wu D, Shi M (2018). Menin Deficiency leads to depressive-like behaviors in mice by modulating astrocyte-mediated neuroinflammation. Neuron.

[21] Zhang Z, Wang M, Xie D, Huang Z, Zhang L, Yang Y, Ma D, Li W, Zhou Q, Yang YG (2018). METTL3-mediated N(6)-methyladenosine mRNA modification enhances long-term memory consolidation. Cell Res.

[22] Pan RY, He L, Zhang J, Liu X, Liao Y, Gao J, Liao Y, Yan Y, Li Q, Zhou X (2022). Positive feedback regulation of microglial glucose metabolism by histone H4 lysine 12 lactylation in Alzheimer’s disease. Cell Metab.

[23] Sun D, Melegari M, Sridhar S, Rogler CE, Zhu L (2006). Multi-miRNA hairpin method that improves gene knockdown efficiency and provides linked multi-gene knockdown. Biotechniques.

[24] Rosario AM, Cruz PE, Ceballos-Diaz C, Strickland MR, Siemienski Z, Pardo M, Schob KL, Li A, Aslanidi GV, Srivastava A (2016). Microglia-specific targeting by novel capsid-modified AAV6 vectors. Mol Ther Methods Clin Dev.

[25] Wang X, Wang Y, Xie F, Song ZT, Zhang ZQ, Zhao Y, Wang SD, Hu H, Zhang YS, Qian LJ (2022). Norepinephrine promotes glioma cell migration through up-regulating the expression of Twist1. BMC Cancer.

[26] Zhao J, Wang C, Zhang X, Li J, Liu Y, Pan X, Zhu L, Chen D, Xie T (2022). Cell membrane coated electrochemical sensor for kinetic measurements of GLUT transport. Anal Chim Acta.

[27] Chan DA, Sutphin PD, Nguyen P, Turcotte S, Lai EW, Banh A, Reynolds GE, Chi JT, Wu J, Solow-Cordero DE (2011). Targeting GLUT1 and the Warburg effect in renal cell carcinoma by chemical synthetic lethality. Sci Transl Med.

[28] Sun ZW, Wang X, Zhao Y, Sun ZX, Wu YH, Hu H, Zhang L, Wang SD, Li F, Wei AJ (2023). Blood-brain barrier dysfunction mediated by the EZH2-Claudin-5 axis drives stress-induced TNF-α infiltration and depression-like behaviors. Brain Behav Immun.

[29] Wang YL, Han QQ, Gong WQ, Pan DH, Wang LZ, Hu W, Yang M, Li B, Yu J, Liu Q (2018). Microglial activation mediates chronic mild stress-induced depressive- and anxiety-like behavior in adult rats. J Neuroinflammation.

[30] Zheng S, Zhang Z, Qu Y, Zhang X, Guo H, Shi X, Cai M, Cao C, Hu Z, Liu H (2019). Radiopharmaceuticals and Fluorescein Sodium Mediated Triple-Modality Molecular Imaging allows precise image-guided tumor surgery. Adv Sci (Weinh).

[31] Vonderhaar EP, Barnekow NS, McAllister D, McOlash L, Eid MA, Riese MJ, Tarakanova VL, Johnson BD, Dwinell MB (2021). STING activated Tumor-intrinsic type I Interferon Signaling promotes CXCR3 Dependent Antitumor Immunity in Pancreatic Cancer. Cell Mol Gastroenterol Hepatol.

[32] Agorastos A, Chrousos GP (2022). The neuroendocrinology of stress: the stress-related continuum of chronic disease development. Mol Psychiatry.

[33] Wang L, Pavlou S, Du X, Bhuckory M, Xu H, Chen M (2019). Glucose transporter 1 critically controls microglial activation through facilitating glycolysis. Mol Neurodegener.

[34] Dresselhaus EC, Meffert MK (2019). Cellular specificity of NF-kappaB function in the nervous system. Front Immunol.

[35] Heneka MT, Carson MJ, El Khoury J, Landreth GE, Brosseron F, Feinstein DL, Jacobs AH, Wyss-Coray T, Vitorica J, Ransohoff RM (2015). Neuroinflammation in Alzheimer’s disease. Lancet Neurol.

[36] Liu YZ, Wang YX, Jiang CL (2017). Inflammation: the common pathway of stress-related diseases. Front Hum Neurosci.

[37] Abbaszade-Cheragheali A, Kakhki S, Khatibi SR, Hosseini M, Navari F, Beheshti F. Feeding crocin ameliorate cognitive dysfunction, oxidative stress and neuroinflammation induced by unpredictable chronic mild stress in rats. Inflammopharmacology; 2023. 10.1007/s10787-023-01250-9 37261629

[38] Wang L, Peng G, Chen L, Guo M, Wang B, Zhang Y, Zhou J, Zhong M, Ye J (2023). Icariin reduces cognitive dysfunction induced by surgical trauma in aged rats by inhibiting hippocampal neuroinflammation. Front Behav Neurosci.

[39] Picard K, St-Pierre MK, Vecchiarelli HA, Bordeleau M, Tremblay ME (2021). Neuroendocrine, neuroinflammatory and pathological outcomes of chronic stress: a story of microglial remodeling. Neurochem Int.

[40] Janani R, Anitha RE, Divya P, Chonche M, Baskaran V (2022). Astaxanthin ameliorates hyperglycemia induced inflammation via PI3K/Akt-NF-kappaB signaling in ARPE-19 cells and diabetic rat retina. Eur J Pharmacol.

[41] Lai CH, Van Thao D, Tsai BC, Hsieh DJ, Chen MY, Kuo WW, Kuo CH, Lu SY, Liao SC, Lin KH (2023). Insulin-like growth factor II receptor alpha overexpression in heart aggravates hyperglycemia-induced cardiac inflammation and myocardial necrosis. Environ Toxicol.

[42] Charles-Messance H, Sheedy FJ (2021). Train to lose: Innate Immune Memory in Metaflammation. Mol Nutr Food Res.

[43] Roberts BL, Karatsoreos IN (2021). Brain-body responses to chronic stress: a brief review. Fac Rev.

[44] Onyango IG, Jauregui GV, Carna M, Bennett JP Jr., Stokin GB. Neuroinflammation in Alzheimer’s Disease. Biomedicines 2021, 9(5). 10.3390/biomedicines9050524 PMC8150909 34067173

[45] Hotamisligil GS (2017). Inflammation, metaflammation and immunometabolic disorders. Nature.

[46] Wu H, Wang M, Li X, Shao Y (2021). The Metaflammatory and Immunometabolic Role of Macrophages and Microglia in Diabetic Retinopathy. Hum Cell.

[47] Zhang D, Jin W, Wu R, Li J, Park SA, Tu E, Zanvit P, Xu J, Liu O, Cain A (2019). High glucose intake exacerbates autoimmunity through reactive-oxygen-species-mediated TGF-beta cytokine activation. Immunity.

[48] van Niekerk G, Davis T, Patterton HG, Engelbrecht AM (2019). How does inflammation-Induced Hyperglycemia cause mitochondrial dysfunction in Immune cells?. BioEssays.

[49] Sanghez V, Razzoli M, Carobbio S, Campbell M, McCallum J, Cero C, Ceresini G, Cabassi A, Govoni P, Franceschini P (2013). Psychosocial stress induces hyperphagia and exacerbates diet-induced insulin resistance and the manifestations of the metabolic syndrome. Psychoneuroendocrinology.

[50] Ishibashi K, Wagatsuma K, Ishiwata K, Ishii K (2016). Alteration of the regional cerebral glucose metabolism in healthy subjects by glucose loading. Hum Brain Mapp.

[51] Mogyorósi A, Ziyadeh FN (1999). GLUT1 and TGF-beta: the link between hyperglycaemia and diabetic nephropathy. Nephrology, dialysis, transplantation: official publication of the European Dialysis and Transplant Association. Eur Ren Association.

[52] Liu Z, Hayashi H, Matsumura K, Ogata Y, Sato H, Shiraishi Y, Uemura N, Miyata T, Higashi T, Nakagawa S (2023). Hyperglycaemia induces metabolic reprogramming into a glycolytic phenotype and promotes epithelial-mesenchymal transitions via YAP/TAZ-Hedgehog signalling axis in pancreatic cancer. Br J Cancer.

[53] Akman CI, Engelstad K, Hinton VJ, Ullner P, Koenigsberger D, Leary L, Wang D, De Vivo DC (2010). Acute hyperglycemia produces transient improvement in glucose transporter type 1 deficiency. Ann Neurol.

[54] Orihuela R, McPherson CA, Harry GJ (2016). Microglial M1/M2 polarization and metabolic states. Br J Pharmacol.

[55] Solmi M, Radua J, Olivola M, Croce E, Soardo L, Salazar de Pablo G, Il Shin J, Kirkbride JB, Jones P, Kim JH (2022). Age at onset of mental disorders worldwide: large-scale meta-analysis of 192 epidemiological studies. Mol Psychiatry.

[56] Borsini A, Giacobbe J, Mandal G, Boldrini M (2023). Acute and long-term effects of adolescence stress exposure on rodent adult hippocampal neurogenesis, cognition, and behaviour. Mol Psychiatry.

[57] Wu MV, Shamy JL, Bedi G, Choi CW, Wall MM, Arango V, Boldrini M, Foltin RW, Hen R (2014). Impact of social status and antidepressant treatment on neurogenesis in the baboon hippocampus. Neuropsychopharmacology: Official Publication Am Coll Neuropsychopharmacol.

[58] Adhikari N, Basi DL, Carlson M, Mariash A, Hong Z, Lehman U, Mullegama S, Weir EK, Hall JL (2011). Increase in GLUT1 in smooth muscle alters vascular contractility and increases inflammation in response to vascular injury. Arterioscler Thromb Vasc Biol.

[59] Chen Z, Dudek J, Maack C, Hofmann U (2021). Pharmacological inhibition of GLUT1 as a new immunotherapeutic approach after myocardial infarction. Biochem Pharmacol.

[60] Cornwell A, Ziolkowski H, Badiei A. Glucose transporter Glut1-Dependent metabolic reprogramming regulates Lipopolysaccharide-Induced inflammation in RAW264.7 macrophages. Biomolecules 2023, 13(5). 10.3390/biom13050770 PMC10216519 37238640

[61] Zhang T, Ouyang H, Mei X, Lu B, Yu Z, Chen K, Wang Z, Ji L (2019). Erianin alleviates diabetic retinopathy by reducing retinal inflammation initiated by microglial cells via inhibiting hyperglycemia-mediated ERK1/2-NF-kappaB signaling pathway. FASEB J.

[62] Bisht K, Sharma K, Tremblay ME (2018). Chronic stress as a risk factor for Alzheimer’s disease: roles of microglia-mediated synaptic remodeling, inflammation, and oxidative stress. Neurobiol Stress.

[63] Freemerman AJ, Johnson AR, Sacks GN, Milner JJ, Kirk EL, Troester MA, Macintyre AN, Goraksha-Hicks P, Rathmell JC, Makowski L (2014). Metabolic reprogramming of macrophages: glucose transporter 1 (GLUT1)-mediated glucose metabolism drives a proinflammatory phenotype. J Biol Chem.

[64] Wu J, Li J, Gaurav C, Muhammad U, Chen Y, Li X, Chen J, Wang Z (2021). CUMS and dexamethasone induce depression-like phenotypes in mice by differentially altering gut microbiota and triggering macroglia activation. Gen Psychiatry.

[65] Xiu J, Li J, Liu Z, Wei H, Zhu C, Han R, Liu Z, Zhu W, Shen Y, Xu Q (2022). Elevated BICD2 DNA methylation in blood of major depressive disorder patients and reduction of depressive-like behaviors in hippocampal Bicd2-knockdown mice. Proc Natl Acad Sci U S A.

[66] Głombik K, Detka J, Góralska J, Kurek A, Solnica B, Budziszewska B (2020). Brain metabolic alterations in rats showing Depression-Like and obesity phenotypes. Neurotox Res.

[67] Miller AH, Raison CL (2016). The role of inflammation in depression: from evolutionary imperative to modern treatment target. Nat Rev Immunol.

[68] Wu CS, Hsu LY, Wang SH (2020). Association of depression and diabetes complications and mortality: a population-based cohort study. Epidemiol Psychiatric Sci.

[69] Li X, Qiu W, Li N, Da X, Ma Q, Hou Y, Wang T, Song M, Chen J (2020). Susceptibility to hyperglycemia in rats with stress-Induced Depressive-Like Behavior: involvement of IL-6 mediated glucose Homeostasis Signaling. Front Psychiatry.

[70] Li ZR, Han YS, Liu Z, Zhao HQ, Liu J, Yang H, Wang YH (2021). GR/NF-κB signaling pathway regulates hippocampal inflammatory responses in diabetic rats with chronic unpredictable mild stress. Eur J Pharmacol.

[71] Li M, Li C, Yu H, Cai X, Shen X, Sun X, Wang J, Zhang Y, Wang C (2017). Lentivirus-mediated interleukin-1β (IL-1β) knock-down in the hippocampus alleviates lipopolysaccharide (LPS)-induced memory deficits and anxiety- and depression-like behaviors in mice. J Neuroinflammation.

[72] Pan Y, Chen XY, Zhang QY, Kong LD (2014). Microglial NLRP3 inflammasome activation mediates IL-1β-related inflammation in prefrontal cortex of depressive rats. Brain Behav Immun.

