# Supplementary material for: GLUT1-mediated microglial proinflammatory activation contributes to the development of stress-induced spatial learning and memory dysfunction in mice
Source: Cell Biosci. 2024 Apr 16;14:48. doi: 10.1186/s13578-024-01229-1 (PMC11020476; doi:10.1186/s13578-024-01229-1)
Supplement: Supplementary file 1 — Supplementary Material 1 [file 13578_2024_1229_MOESM1_ESM.docx]

**GLUT1-mediated Microglial Proinflammatory Activation Contributes to the Development of Stress-induced Spatial Learning and Memory Dysfunction in Mice**

Xue Wang ^a,^ **^*^**, Yuhan Wu ^a,^ **^*^**, Yingrui Tian ^a, b,^ **^*^**, Hui Hu ^a^, Yun Zhao ^a^, Binghua Xue ^a^, Zhaowei Sun ^a^, Aijun Wei ^a^, Fang Xie ^a,^ **^#^**, Ling-Jia Qian ^a, #^

^a^ Beijing Institute of Basic Medical Sciences, Academy of Military Medical Sciences, Beijing, China, 100850.

^b^ Centers for Disease Control and Prevention, Jiulongpo District, Chongqing, China, 400050.

**^*^** The authors contributed equally to this work.

**^#^ Correspondence to:** Lingjia Qian, MD, PhD and Fang Xie, PhD

Beijing Institute of Basic Medical Sciences, Academy of Military Medical Sciences, #27 Taiping Road, Haidian, Beijing, China.

Tel: +86-10-66931393.

E-mail: stressqian@163.com (Lingjia Qian), vancoxie@sina.com (Fang Xie)

**Methods**

**Intrahippocampal injection of** **lipopolysaccharide (LPS)**

Healthy male C57BL/6J mice were anesthetized with 5% chloral hydrate (100 μl/10 g body weight) by intraperitoneal injection and then placed on a stereotaxic apparatus. Bilateral small holes were drilled into the skull, and the injection site was located into the hippocampus using coordinates at -2.46 mm caudal to Bregma, ±1.7 mm from midline, and -2.0 mm deep from the skull. LPS (500 μg/kg, dissolved in saline) was delivered via a Hamilton syringe at a rate of 0.1 µl/min. After 24 h, the mice were sacrificed.

**Supplementary Figures and Figure Legends**

**
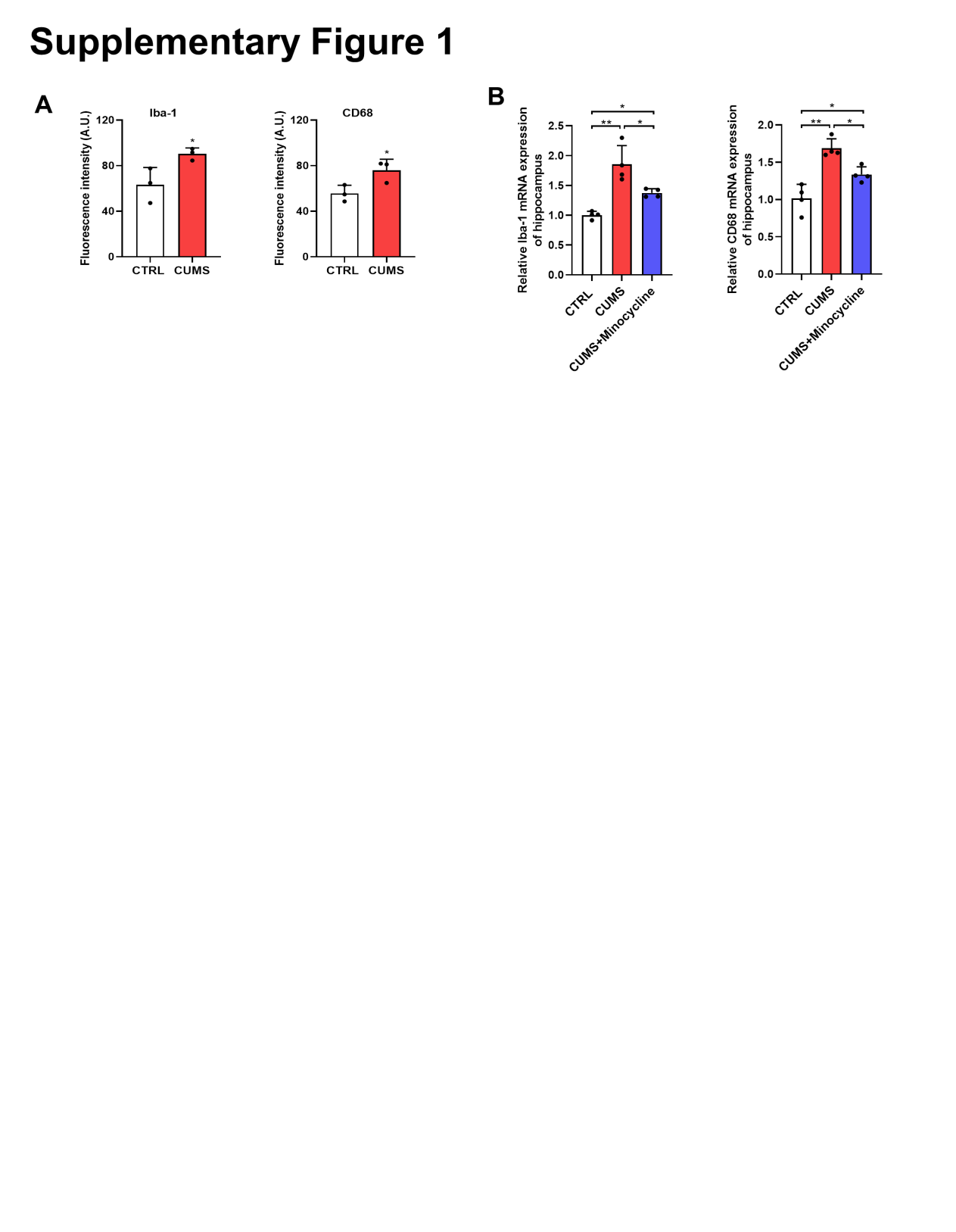
**

**Supplementary Figure 1.**

(A) Quantification of the fluorescence intensity of Iba1 and CD68 in the hippocampal sections of CTRL and CUMS mice (n = 3, Student’s t-test). (B) qRT-PCR assays monitoring the expression of Iba1 and CD68 in hippocampal samples from CTRL, CUMS, and CUMS + Mino mice (n = 4, One-way ANOVA with Tukey's post hoc test). * p < 0.05, ** p < 0.01.

**
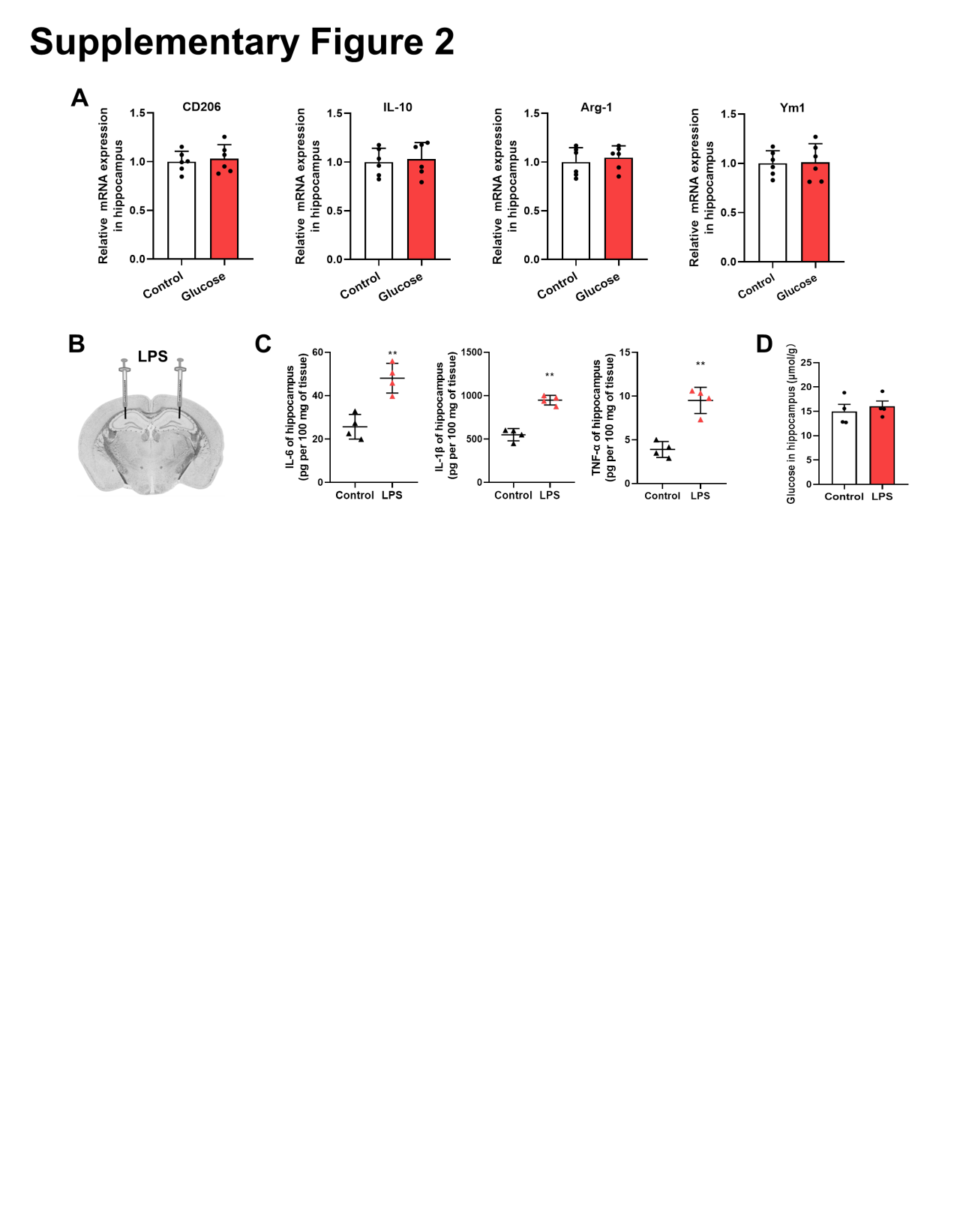
**

**Supplementary Figure 2.**

(A) qRT-PCR assays monitoring the expression of anti-inflammatory phenotype markers, CD206, IL-10, Arg-1, and Ym1 in hippocampal samples from glucose-infused and control mice (n = 6, Student’s t-test). (B) Illustration of bilateral LPS injections into the mouse hippocampus. Mice injected with saline were used as a control. (C) Levels of IL-6, IL-1β, and TNF-α in hippocampus lysates from control and LPS-injected mice were measured by ELISA (n = 4, Student’s t-test). (D) Glucose levels in hippocampus lysates from control and LPS-injected mice (n = 4, Student’s t-test). ** p < 0.01.**
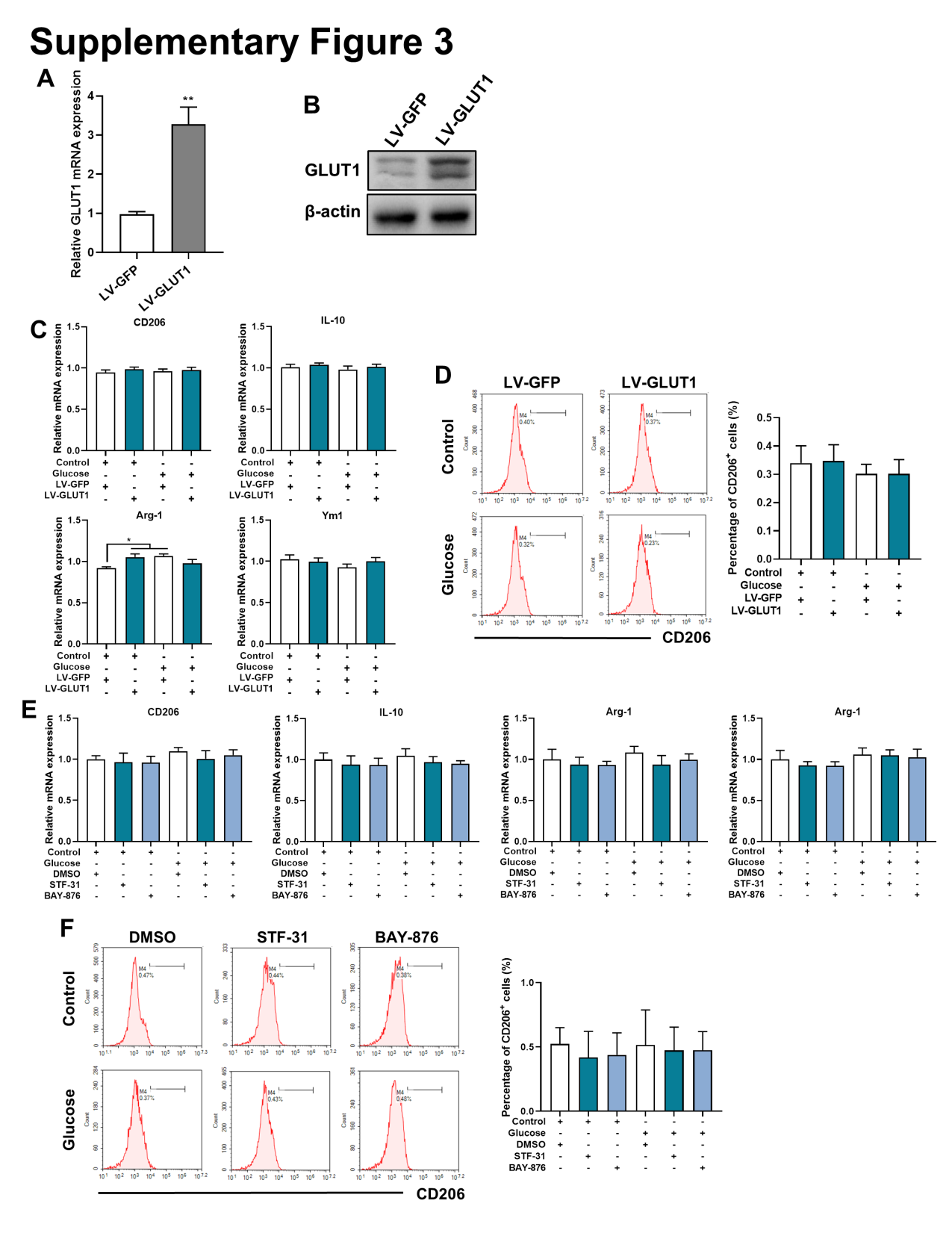
**

**Supplementary Figure 3.**

(A) Stable BV2 cell lines expressing GFP (LV-GFP) or GLUT1 (LV-GLUT1) were established using lentivirus. The overexpression of GLUT1 was validated by qRT-PCR (n = 3, Student’s t-test). (B) The expression of GLUT1 in LV-GFP and LV-GLUT1 cells was examined by western blotting. (C) qRT-PCR assays monitoring the expression of anti-inflammatory phenotype markers, CD206, IL-10, Arg-1, and Ym1 in BV2-LV-GFP/LV-GLUT1 cells with or without glucose treatment (n = 6, One-way ANOVA with Tukey's post hoc test). (D) Flow cytometry analysis of CD206^+^ populations in BV2-LV-GFP/LV-GLUT1 cells under control or glucose conditions. Representative images (left); quantified result (right, n = 5, One-way ANOVA with Tukey's post hoc test). (E) qRT-PCR assays monitoring the expression of anti-inflammatory phenotype markers, CD206, IL-10, Arg-1, and Ym1 in BV2 cells exposed to STF-31 or BAY-876 with or without glucose treatment (n = 6, One-way ANOVA with Tukey's post hoc test). (F) Flow cytometry analysis of CD206^+^ populations in BV2 cells exposed to STF-31 or BAY-876 under control or glucose conditions. Representative images (left); quantified result (right, n = 5, One-way ANOVA with Tukey's post hoc test). * p < 0.05, ** p < 0.01.


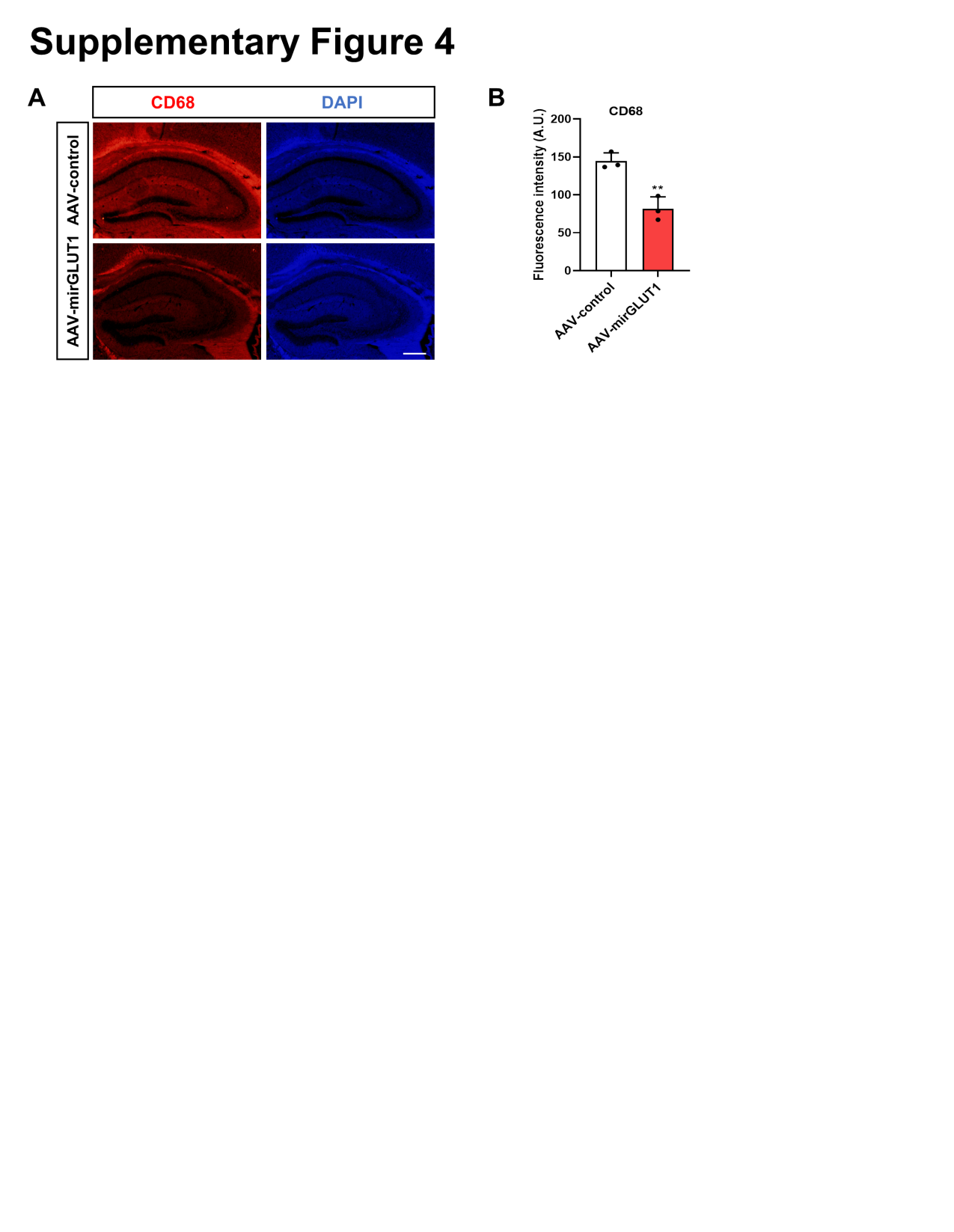


**Supplementary Figure 4.**

(A) Representative images of IF staining of hippocampal sections from AAV-mirGLUT1 or AAV-control injected mice. CD68 red; DAPI, blue. Scale bar, 200 μm. (B) Quantification of the fluorescence intensity of CD68 in the hippocampal sections of AAV-mirGLUT1 or AAV-control injected mice (n = 3, Student’s t-test). ** p < 0.01.

**
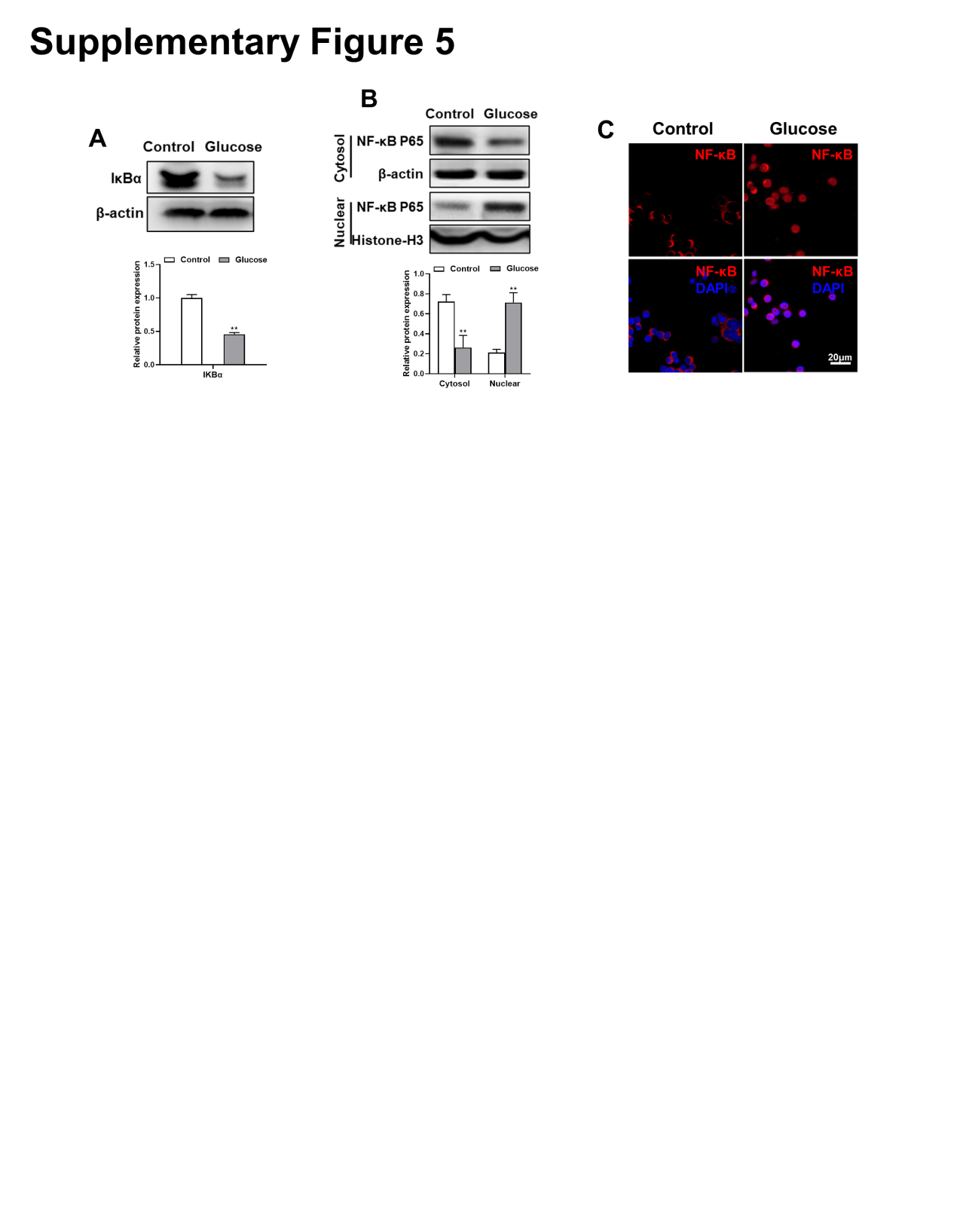
**

**Supplementary Figure 5.**

(A) Levels of IκBα proteins in glucose-treated BV2 cells and control cells were determined by western blotting, and the quantified result relative to β-actin is shown (n=3, Student’s t-test). (B) Cytosolic and nuclear fractions of glucose-treated BV2 and control cells were isolated, and the level of NF-κB p65 protein was evaluated by western blotting. The quantified result is shown (n=3, Student’s t-test). (C) NF-κB nuclear translocation in glucose-treated BV2 cells and control cells was analyzed by IF staining. Representative images are shown. Scale bar, 20 μm. ** p < 0.01.

**Supplementary Tables**

**Supplementary Table 1. Primer List.**

| Gene | Primer sequence | |
| --- | --- | --- |
| CD86 | Forward primer (5’-3’) | CTTACGGAAGCACCCACGAT |
|  | Reverse primer (5’-3’) | TGTAAATGGGCACGGCAGAT |
| IL-6 | Forward primer (5’-3’) | GCTACCTGGAGTACATGAAGAACA |
|  | Reverse primer (5’-3’) | GGTCCTTAGCCACTCCTTCTG |
| IL-1β | Forward primer (5’-3’) | TGTGCTCCTTGTCAACAGCG |
|  | Reverse primer (5’-3’) | TTTCAATTCTGTGGCCTGCTTG |
| TNF-α | Forward primer (5’-3’) | CCCACGTCGTAGCAAACCA |
|  | Reverse primer (5’-3’) | ACAAGGTACAACCCATCGGC |
| CD206 | Forward primer (5’-3’) | GTGGGGACCTGGCAAGTATC |
|  | Reverse primer (5’-3’) | CACTGGGGTTCCATCACTCC |
| IL-10 | Forward primer (5’-3’) | GCTCCAAGACCAAGGTGTCTA |
|  | Reverse primer (5’-3’) | ACGAGGTTTTCCAAGGAGTTGT |
| Arg1 | Forward primer (5’-3’) | CTTGCGAGACGTAGACCCTG |
|  | Reverse primer (5’-3’) | CTTCCTTCCCAGCAGGTAGC |
| Ym1 | Forward primer (5’-3’) | GCCCACCAGGAAAGTACACA |
|  | Reverse primer (5’-3’) | CTTGAGCCACTGAGCCTTCA |
| Iba-1 | Forward primer (5’-3’) | CTTGAAGCGAATGCTGGAGAA |
|  | Reverse primer (5’-3’) | GGCAGCTCGGAGATAGCTTT |
| CD68 | Forward primer (5’-3’) | TGTCTGATCTTGCTAGGACCG |
|  | Reverse primer (5’-3’) | GAGAGTAACGGCCTTTTTGTGA |
| GLUT1 | Forward primer (5’-3’) | CTCACCACGCTTTGGTCTCT |
|  | Reverse primer (5’-3’) | CCCAGTTTGGAGAAGCCCAT |
| GLUT2 | Forward primer (5’-3’) | ATCACCGGAACCTTGGCTTT |
|  | Reverse primer (5’-3’) | CAGCTTTCCGGTCATCCAGT |
| GLUT3 | Forward primer (5’-3’) | CCTACCAAGTGAGGGACTGC |
|  | Reverse primer (5’-3’) | GGCCCAGGATCAGCATTTCA |
| GLUT4 | Forward primer (5’-3’) | TTGGCTCCCTTCAGTTTGGC |
|  | Reverse primer (5’-3’) | CGTAGTGAGGGTGCCTTGTG |
| GLUT5 | Forward primer (5’-3’) | TGCCTTTGGCTCATCCTTCC |
|  | Reverse primer (5’-3’) | TAAAGCCCCCAAAGGGGAAC |
| GLUT6 | Forward primer (5’-3’) | TCTGACCCAGCACTACACCT |
|  | Reverse primer (5’-3’) | CATCAGGGGGTGCAATCTCA |
| GLUT7 | Forward primer (5’-3’) | TGACTACCCTAAGTGCTGCC |
|  | Reverse primer (5’-3’) | CCTGAAAGACAGGACCCCAT |
| GLUT8 | Forward primer (5’-3’) | GCCCCTCATCATCGGCATTT |
|  | Reverse primer (5’-3’) | ACCTGGATTATGCCCACAGT |
| GLUT9 | Forward primer (5’-3’) | TTCCCTGATGTGTGACACCG |
|  | Reverse primer (5’-3’) | AGGCCTTGATATACGGCGTG |
| GLUT10 | Forward primer (5’-3’) | GAAGCTCGGGTTTGCTTGTG |
|  | Reverse primer (5’-3’) | CTCAAGGATGGGACTGGTGG |
| GLUT11 | Forward primer (5’-3’) | CGGACCTGCCTCTCACGCAATGG |
|  | Reverse primer (5’-3’) | CACCTGTCCCATCACGATCC |
| GLUT12 | Forward primer (5’-3’) | AAACACAGAGGGCCCCAATC |
|  | Reverse primer (5’-3’) | CAACCATTTCCTGCTCGTGG |
| β-actin | Forward primer (5’-3’) | CGATGCCCTGAGGCTCTTTT |
|  | Reverse primer (5’-3’) | GAGGTCTTTACGGATGTCAACG |

**Supplementary Table 2. Antibody List.**

| Antigens | Manufacturer | Application |
| --- | --- | --- |
| Iba-1 | Abcam, USA | 1:200 for IF |
| CD68 | Abcam, USA | 1:100 for IF |
| GLUT1 | Proteintech, USA | 1:500 for WB |
| GLUT1 | Abclonal Technology, China | 1:1000 for IF |
| IkBα | Abclonal Technology, China | 1:1000 for WB |
| NF-kB p65 | Abclonal Technology, China | 1:1000 for WB  1:100 for IF |
| β-actin | ZSGB-bio, China | 1:1000 for WB |
| Histone H3 | Abclonal Technology, China | 1:2000 for WB |

WB, western blotting; IF, immunofluorescence
